# Functional Characterization of the Osteoarthritis Susceptibility Mapping to *CHST11*—A Bioinformatics and Molecular Study

**DOI:** 10.1371/journal.pone.0159024

**Published:** 2016-07-08

**Authors:** Louise N. Reynard, Madhushika Ratnayake, Mauro Santibanez-Koref, John Loughlin

**Affiliations:** 1 Musculoskeletal Research Group, Institute of Cellular Medicine, Newcastle University, Newcastle upon Tyne, United Kingdom; 2 Institute of Genetic Medicine, Newcastle University, Newcastle upon Tyne, United Kingdom; University of Texas Rio Grande Valley, UNITED STATES

## Abstract

The single nucleotide polymorphism (SNP) rs835487 is associated with hip osteoarthritis (OA) at the genome-wide significance level and is located within *CHST11*, which codes for carbohydrate sulfotransferase 11. This enzyme post-translationally modifies proteoglycan prior to its deposition in the cartilage extracellular matrix. Using bioinformatics and experimental analyses, our aims were to characterise the rs835487 association signal and to identify the causal functional variant/s. Database searches revealed that rs835487 resides within a linkage disequilibrium (LD) block of only 2.7 kb and is in LD (r^2^ ≥ 0.8) with six other SNPs. These are all located within intron 2 of *CHST11*, in a region that has predicted enhancer activity and which shows a high degree of conservation in primates. Luciferase reporter assays revealed that of the seven SNPs, rs835487 and rs835488, which have a pairwise r^2^ of 0.962, are the top functional candidates; the haplotype composed of the OA-risk conferring G allele of rs835487 and the corresponding T allele of rs835488 (the G-T haplotype) demonstrated significantly different enhancer activity relative to the haplotype composed of the non-risk A allele of rs835487 and the corresponding C allele of rs835488 (the A-C haplotype) (*p* < 0.001). Electrophoretic mobility shift assays and supershifts identified several transcription factors that bind more strongly to the risk-conferring G and T alleles of the two SNPs, including SP1, SP3, YY1 and SUB1. *CHST11* was found to be upregulated in OA versus non-OA cartilage (*p* < 0.001) and was expressed dynamically during chondrogenesis. Its expression in adult cartilage did not however correlate with rs835487 genotype. Our data demonstrate that the OA susceptibility is mediated by differential protein binding to the alleles of rs835487 and rs835488, which are located within an enhancer whose target may be *CHST11* during chondrogenesis or an alternative gene.

## Introduction

Osteoarthritis (OA) is a common disease of older individuals that is characterized by the focal loss of articular cartilage [1]. This loss usually occurs gradually over many years and typically results in chronic pain and severely impaired joint function by the sixth or seventh decade of life. There is accumulating evidence of increased mortality associated with the disease, which is the result of secondary cardiovascular events arising from lower patient activity [2]. Other joint tissues also show OA-associated changes, but alteration to normal cartilage function is pivotal [3].

OA is polygenic and unlike many other common arthritic diseases, there are no OA risk-conferring loci of large singular impact [4]. A number of genome-wide and candidate-gene based studies have reported OA association signals that exceed or are close to the genome-wide significance threshold (reviewed in [5]). Many of these signals demonstrate association only in a particular joint type; for example, hips but not knees. Stratification by joint has therefore proven essential in identifying OA association signals and highlights that, at the genetic level, OA aetiology is not uniform across the skeleton.

The most powerful OA genome-wide association scan (GWAS) so far performed is the arcOGEN study, which identified five signals at the genome-wide significance threshold and three just below [6]. These signals were detected following the direct typing and imputation of approximately 1.4 million autosomal single nucleotide polymorphisms (SNPs). Whilst several of the arcOGEN signals encompass genes lacking a prior known role in joint tissue biology, some do and one that fits into this category is on chromosome 12q23 and marked by rs835487. This SNP is associated with hip OA in males and females at the genome-wide significance level, with a *p*-value of 1.64 x 10^−8^. It is located within intron 2 of the carbohydrate sulfotransferase 11 gene *CHST11*, which encodes an enzyme that catalyses the transfer of sulfate groups to chondroitin to form chondroitin-4-sulfate. This is a major component of glycosaminoglycan (GAG) chains, which bind to a core protein in the cartilage extracellular matrix to form proteoglycans that can modulate the access of growth factors to the cartilage chondrocyte cells [7].

Following the mapping of a susceptibility locus, it is important to determine the molecular mechanism linking genetic variation with disease risk. One step in the characterisation of the signal is the identification of the causal variant. In a genomic region where the associated variant is in high (r^2^ > 0.8) to perfect (r^2^ = 1) linkage disequilibrium (LD) with other SNPs, this requires direct functional analysis of these correlating SNPs using relevant *in silico* and *in vitro* assays. The rs835487 association signal does not correlate with any non-synonymous SNPs, which suggests that the functional effect of this OA signal is on gene expression rather than via an amino acid substitution. There are several published examples in OA of risk-conferring SNPs whose effects are mediated by modulating gene expression, including the *GDF5* SNP rs143383, the *DIO2* SNP rs225014, and the *ALDH1A2* SNP rs3204689 [8–11].

We therefore hypothesised that the 12q23 OA association signal acts by modulating gene expression and we set out to determine if this was the case by functionally investigating rs835487 and the SNPs in LD with it. We employed bioinformatics analyses and a range of experimental techniques to determine which, if any, of the SNPs modulated gene expression at the allelic level, including luciferase reporter assays, electrophoretic mobility shift assays, supershifts and allelic expression imbalance studies. In so doing we examined a number of relevant cell types and tissue, namely transformed cell lines, mesenchymal stem cells undergoing chondrogenic differentiation, and cartilage from OA patients.

## Materials and Methods

### Identification of SNPs in LD with rs835487

SNPs with an r^2^ of over 0.8 with the OA marker SNP rs835487 in the European population (EUR; similar to the individuals used in the arcOGEN study) were identified using the LDlink bioinformatics tool (http://analysistools.nci.nih.gov/LDlink/; [12]), which uses genotyping data from Phase 3 of the 1000 Genomes Project [13]. The EUR population is composed of Utah residents of northern and western European ancestry (CEU; n = 99), British people from England and Scotland (GBR; n = 91), Finnish individuals (FIN; n = 99), the Iberian population in Spain (IBS; n = 107) and individuals from Toscani in Italy (TSI; n = 107). The major alleles and minor OA-risk alleles for each SNP were identified using dbSNP (http://www.ncbi.nlm.nih.gov/SNP/) and the probabilistic identification of causal SNPs (PICS) scores, which serve to rank SNPs at a given locus as a means of identifying the most likely causal SNP, were calculated using the PICS bioinformatics tool (https://pubs.broadinstitute.org/pubs/finemapping/pics.php; [14]).

### Identification of Sequences Homologous to the Enhancer in Non-Human Mammals

The 2.7 kb genomic region encompassing the human enhancer region was used to interrogate the genomes of other mammals using the UCSC BLAT software (http://genome.ucsc.edu/cgi-bin/hgBlat). In species that had homology to the human enhancer region within intron 2 of the orthologous *CHST11* gene, the homologous regions were aligned to the entire human enhancer sequence as well as to the regions immediately surrounding the seven SNPs individually using the ClustalW Multiple Sequence Alignment Tool (http://www.ebi.ac.uk/Tools/msa/clustalw2/). The UCSC LiftOver tool was used to map the *Chst11* chondrogenic enhancer regions identified by Ohba et al. [15] and Liu and Lefebvre [16] from rat assembly RGSC 5.0/rn5 and mouse assembly NCBI37/mm9 to the human GRCh37/hg19 assembly respectively. Regions of nucleotide conservation between the rodent and human sequences were identified using the NCBI blastn sequence alignment tool that is optimised for ‘somewhat similar sequences’.

### Cloning of pGL3-Promoter Luciferase Reporter Plasmids

The regions chromosome 12:105059890–105061288 (containing the rs835486, rs835487 and rs835488 SNPs), chromosome 12:105062129–105063197 (encompassing the rs835490, rs835491, rs835492 and rs835493 SNPs), chromosome 12:105059891–105060550 (rs835486 only), chromosome 12:105060261–105060975 (rs835487 only) and chromosome 12:105060784–105061471 (rs835488 only) were amplified from heterozygous genomic DNA and cloned into the pGL3-Promoter vector (Promega) upstream of the SV40 Promoter. Two plasmids were created for each region, one with the major haplotype or allele and one with the minor OA-risk haplotype or allele. The different haplotype combinations of the rs835486-rs835487-rs835488 vector were created by site-directed mutagenesis of the AAC haplotype using the Quikchange II Site-Directed Mutagenesis kit (Agilent) according to the manufacturer’s instructions. Sanger sequencing confirmed the sequence and haplotype/genotype of each vector, and the DNA was then purified using the PureYield Plasmid Maxiprep System (Promega). Primer sequences are listed in S1 Table.

### Transfection of Cell Lines

SW1353 human chondrosarcoma cells (ATCC) were seeded at a density of 1.75 x 10^4^ cells per well in a 48-well plate 24 hours prior to transfection. MDA-MB-231 human adenocarcinoma cells (ATCC) were seeded at a density of 2.5 x 10^4^ cells per well in a 48-well plate 48 hours prior to transfection. SW1353 and MDA-MB-231 cells were co-transfected with 500 ng of the appropriate pGL3-promoter vector and 30 ng of pRL-TK *Renilla* control vector (Promega) using TurboFect transfection reagent (Thermo Scientific). After 24 hours, cells were lysed and luciferase and *Renilla* activity measured using the Dual Luciferase Assay System (Promega) according to the manufacturers’ protocol. For each construct, six wells were transfected and at least six independent experiments performed per cell line. The luciferase/renilla ratio of each construct was normalised to the empty pGL3-promoter vector, which was given a ratio of 1. The significance of expression differences were assessed using a Mann-Whitney U test.

### Electrophoretic Mobility Shift Assays (EMSAs)

Nuclear proteins were extracted from SW1353 cells and MDA-MB-231 cells and quantified as described previously [17]. Single-stranded DY-682 labelled oligonucleotides were synthesised (Eurofins MWG Operons), annealed to generate double-stranded probes and diluted to 100 fmol/μl. Binding reactions containing 5 μg of nuclear protein and 200 fmol of probe were performed using the Odyssey EMSA Buffer kit (Licor Biosciences) as previously described [18], with the addition of 0.4% NP40 and 2.5% glycerol to the rs835487 binding reactions. Samples were electrophoresed through a 5% (w/v) polyacrylamide gel and visualised as in Reynard et al. 2014 [18]. Transcription factors predicted to bind over the rs845487 or rs835488 SNPs were identified using MatInspector, PROMO, TESS and TF Search online databases [19,20]. For competition assays, unlabelled competitors identical to the probes or containing the consensus sequence of transcription factors predicted to bind over the SNP were added to the binding reaction in excess (10-, 25-, 50-, and 100-fold for competitors identical to the probes; 1-, 2.5-, 5- and 10-fold for SP1/SP3 competitor to the rs835487 EMSA; 5-, 10-, 25- and 50-fold for SP1/SP3 competitor to the rs835488 EMSA). For supershift assays, 2 μg of rabbit polyclonal antibodies against various transcription factors including anti-SP1 (Santa Cruz Biotechnology; sc-59 X), anti-SP3 (sc-644 X), anti-SUB1 (sc-48778 X), and anti-YY1 rabbit (sc-281 X) were added to the binding reaction, with anti-PAX6 polyclonal antibody being used as an IgG control. The probe and SP1/SP3 consensus competitor sequences are listed in S1 Table.

### Ethics Statement, Cartilage Collection and Nucleic Acid Extraction

The Newcastle and North Tyneside Research Ethics Committee granted ethical approval for the collection of cartilage from patients undergoing hip or knee replacement for primary OA, or hip replacement resulting from a neck of femur (NOF) fracture (REC reference number 14/NE/1212). Each donor provided informed consent. The project was discussed with the donor verbally by a trained research nurse and if the donor agreed to participate written consent was then taken. This consent procedure was approved by the ethics committee and the written consent was then filed by the consenting nurse. OA status was confirmed using pre-operative records and all OA patients had full thickness cartilage lesions. For all patients, macroscopically normal articular cartilage was collected [21,22]. For the OA patients, cartilage was collected distal to the OA lesion and these patients were screened to exclude all cases of skeletal or developmental dysplasia, inflammatory arthritis and post-traumatic arthritis. Cartilage samples were collected within four hours of surgery, snap-frozen in liquid nitrogen and stored at -80°C until nucleic acid extraction. The frozen tissue was ground and RNA and DNA extracted using the Omega EZNA Total DNA/RNA isolation kit (R6731-02; Omega Bio-Tek), as previously described [21].

### Gene Expression, Genotyping and AEI Analysis

One microgram of total RNA was reverse transcribed using the SuperScript First-Strand Synthesis System (Invitrogen), and the cDNA diluted 1:20 in DEPC water. *CHST11* expression was assessed by quantitative real-time RT-PCR (qRT-PCR), with three PCR replicates performed per sample. mRNA expression levels were normalised to *18s*, *GAPDH* and *HPRT1* housekeeping genes using the calculation 2^-ΔCt. The rs835487 and rs2463018 SNPs were genotyped by restriction fragment length polymorphism (RFLP) assays using 20 ng of DNA. Allelic expression imbalance (AEI) analysis was assessed by pyrosequencing [23] and was performed on cartilage DNA and mRNA from individuals heterozygous for the rs2463018 SNP present in the 3ʹUTR of *CHST11*. Three DNA and cDNA replicates were performed per sample and the mRNA allelic ratio normalised to the DNA ratio (representing the 1:1 ratio between alleles) for the same sample. The qRT-PCR, RFLP and pyrosequencing primer sequences are listed in S1 Table.

### Chondrogenic Differentiation of MSCs

Human iliac crest-derived mesenchymal stem cells (MSCs) from three donors aged 22–24 years (Lonza Biosciences) were subjected to Transwell chondrogenic differentiation as described by Murdoch et al. [34] and Barter et al. [35]. Cartilage discs were harvested after 3, 7 and 14 days and RNA extracted from the ground tissue using the Trizol/chloroform method (Invitrogen). cDNA was synthesised as for cartilage samples and subjected to qRT-PCR, as above.

## Results

### The OA Susceptibility Maps to a Conserved Region Predicted to Act as an Enhancer

**Fig 1:**
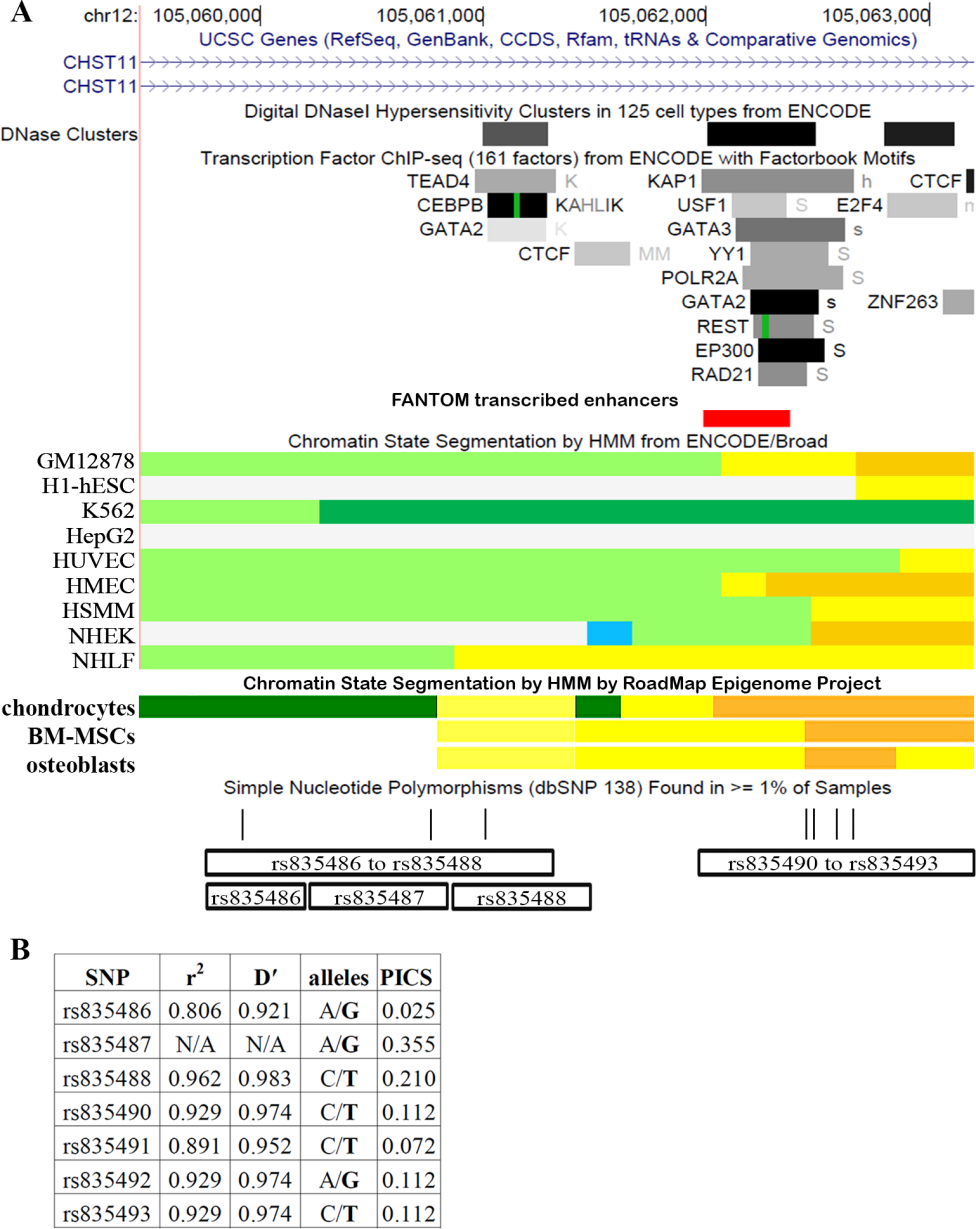
The OA risk locus at chromosome 12q23 maps to a predicted enhancer element. (**A**) UCSC genome browser view of the OA locus with the DNase1 Hypersensitivity, Transcription Factor ChIP-seq, and chromatin state segmentation tracks from the ENCODE project [24], the chromatin state tracks for bone-marrow derived mesenchymal stem cells (BM-MSCs), *in vitro* differentiated chondrocytes and primary osteoblasts from the RoadMap Epigenome project [25], enhancer transcripts from the FANTOM 5 consortium [26] and the SNP track containing data from dbSNP 138. For the SNP track (“Simple Nucleotide Polymorphisms (dbSNP 138) Found in > = 1% of Samples”), the seven vertical lines mark the positions of, from left to right, rs835486, rs835487, rs835488, rs835490, rs835491, rs835492 and rs835493. rs835487 marks the OA association residing at this locus, with the other six SNPs having a pair-wise r^2^ of > 0.8 with rs835487. The positions of the regions cloned into the pGL3 promoter vector for luciferase studies (Fig 2 and S3 Fig) are indicated by rectangles. (**B**) Table of the seven SNPs within the enhancer region marked by rs835487. The alleles of each SNP are listed, with the OA-associated allele highlighted in bold, and the r^2^ and Dʹ to rs835487 in the European population given for the other six SNPs within this LD block. PICS score (probabilistic identification of causal SNPs) is a probability estimate that the SNP is the causal variant [14]; in an LD block the total PICS score will be 1 with SNPs in highest LD with the association SNP having larger PICS scores.

In order to determine the size of the OA susceptibility region marked by rs835487, we used LDlink [12], which utilises genotype data of 503 Europeans generated by the 1000 Genomes Project [13], to identify SNPs in high LD with rs835487. This identified six additional SNPs in the European (EUR) population (the population used in the arcOGEN study) with an r^2^ ≥ 0.8 with rs835487; all seven SNPs are located within a 2726 bp region of intron 2 of *CHST11* (chromosome 12:105059930–105062656), approximately 210 kb downstream of the transcription start site of the gene (Fig 1A and 1B). This region is flanked by two recombination hotspots located at approximately chromosome 12:105059400 and chromosome 12:105063200 (a 3800 bp region), with LD tailing off either side of these hotspots; the SNP with the highest LD with rs835487 outside of the LD block has an r^2^ of only 0.624 and was genotyped in the arcOGEN study, with a less significant *p*-value. Together, this indicates that one or more of the seven SNPs within the 2.7 kb block mediate the OA association signal marked by rs835487.

Analysis of publically available ENCODE datasets on the UCSC genome browser [24] indicates that the LD region contains several DNaseI hypersensitivity sites (DHSs) and transcription factor binding sites (Fig 1A) that overlap several of the seven SNPs, and has a chromatin signature indicative of enhancer activity in several transformed human cell lines. Furthermore, analysis of chromatin data from the RoadMap Epigenome Project [25] reveals that this region is also predicted to act as an enhancer in bone marrow derived mesenchymal stem cells (BM-MSCs), in *in vitro* differentiated chondrocytes and in primary osteoblasts. Examination of the Promoter Enhancer Slide Selection Tool (PrESSto) database [26] reveals that the LD block produces small bidirectional capped transcripts, a signature of active enhancers, in several human cells and tissues. The region is conserved in eutherian mammals, although it has diverged in the lagomorpha and rodentia lineages (S2 Table, S1 and S2 Figs). ClustalW alignments of the 60 bp region surrounding each of the seven SNPs in the 42 mammalian species listed in S2 Table, revealed that the rs835487 SNP is the most conserved (that is, the region surrounding rs835487 is present in the largest number of species), with the rs835486 SNP region only being present in higher primates (S1 Fig). For rs835487, rs835488 and rs835492, the OA allele is the ancestral allele in mammals and is the major allele within the African (AFR) population, with the newly evolved non-OA allele being the major allele in non-AFR populations [13]. The increased frequency of the non-OA alleles at these SNPs in non-Africans suggests that these alleles may have undergone positive selection, although random genetic drift may also be responsible. Furthermore, using the probabilistic identification of causal SNPs (PICS) algorithm [14], rs835487 is predicted to be the causal OA SNP (Fig 1B).

The mouse and rat orthologues of *CHST11* contain several enhancers, which together form a super-enhancer in proliferating and prehypertrophic chondrocytes of both mesodermal and neural crest origin [15,16]. The *Chst11* super-enhancer is bound by the key chondrogenic transcription factor SOX9 in mouse chondrocytes and by the SOX9/SOX5/SOX6 trio in rat chondrocytes. Binding of the SOX trio to the *Chst11* super-enhancer is thought to regulate expression of this gene in chondrocytes; overexpression of SOX9 leads to a ~2 fold upregulation in *CHST11* expression in human dermal fibroblasts [15], whilst expression of *Chst11* is abolished in mouse growth plate chondrocytes lacking *Sox9* or *Sox5*/*So*x6 [16]. Although there is significant sequence divergence between humans and rodents, one of the rodent enhancers within the *Chst11* chondrocyte super-enhancer maps to a region orthologous to the human OA LD region (S2 Fig). Within this homologous region, the highest conservation between the human, rat and mouse sequences occur at regions bound by SOX9 and SOX5/SOX6 and these conserved regions are also positive for the poised and active enhancer marks H3K27Ac and H3K4me1 in E14.5 mouse limbs.

Together, these observations suggest that the OA susceptibility region acts as a conserved distal enhancer of gene expression.

### The OA Region Regulates Promoter Activity, With the Alleles of rs835487 and rs835488 Having Differential Effects

The divergence in nucleotide sequence between humans and rodents, particularly around the SNP regions, precludes direct analysis of the associated region in commonly used animal models, such as mice. Our functional analysis therefore focussed on human cells. To confirm that the OA susceptibility region acts as an enhancer and to identify which of the seven SNPs is the causal SNP(s), we performed *in vitro* luciferase enhancer assays in SW1353 human chondrosarcoma and MDA-MB-231 human adenocarcinoma cell lines. The enhancer region was cloned as two separate fragments into the pGL3 luciferase vector upstream of a minimal promoter, with the first fragment containing three SNPs (rs835486-rs835487-rs835488) and the second fragment four SNPs (rs835490-rs835491-rs835492-rs835493).

**Fig 2:**
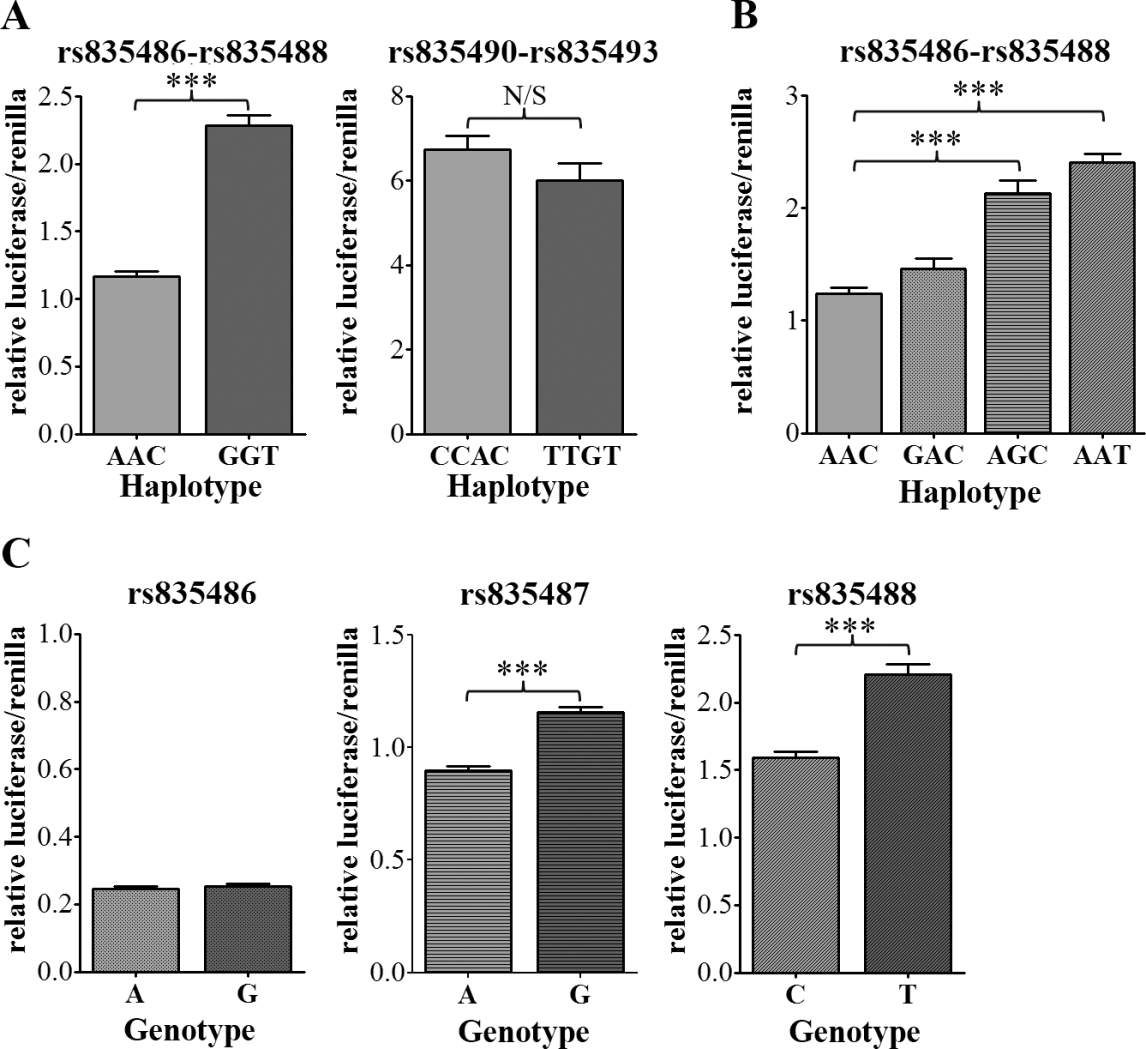
The OA risk locus has haplotype-specific differences in luciferase reporter activity. (**A**) To (**C**) luciferase enhancer assays in the SW1353 human chondrosarcoma cell line using the pGL3 promoter plasmids. The vectors were co-transfected with the pRL-TK Renilla plasmid, and luciferase/renilla values normalised to the empty pGL3-enhancer vector. (**A**) Luciferase activity of the major AAC and OA-associated GGT haplotype of the region containing the rs835486, rs835487 and rs835488 SNPs and luciferase activity of the CCAC and OA-associated TTGT haplotype of the region containing rs835490, rs835491, rs835492 and rs835493. (**B**) Luciferase activity of the rs835486-rs835487-rs835488 vectors containing the AAC, GAC, AGC and AAT haplotypes. Mutating the non-OA allele into the OA-risk allele of rs835486, rs835487 or rs835488, respectively, created the GAC, AGC and AAT vectors. (**C**) Luciferase activity of the rs835486 region (left), rs835487 region (middle) or rs835488 region (right). Data shown is the mean ± standard error of at least four independent experiments, each with five technical repeats. ***p < 0.001, Mann-Whitney *U* test. N/S, not significant. The data points that enabled construction of this figure can be found in S5 Table.

There was significantly higher enhancer activity of the OA-associated minor GGT haplotype of rs835486-rs835487-rs835488 compared to the non-OA AAC haplotype in SW1353 chondrosarcoma (1.96 fold, *p* < 0.001; Fig 2A) and MDA-MB-231 adenocarcinoma cells (1.95 fold, *p* < 0.001; S3A Fig). In SW1353 cells, both haplotypes of this region acted as an enhancer, with higher luciferase activity than the basal empty pGL3 promoter control vector, but in MDA-MB-231 cells, the AAC haplotype acted as a repressor, suggesting the regulatory effect of this region on promoter activity depends on the cellular context. The rs835490-rs835491-rs835492-rs835493 region acted as an enhancer in SW1353 cells but not in MDA-MB-231 cells (Fig 2A and S3B Fig). No significant difference in promoter activity was observed in either cell line between the CCAC and OA-associated TTGT haplotypes for the rs835490-rs835491-rs835492-rs835493 fragment, suggesting the OA susceptibility SNP does not reside in this region.

To identify which of the SNPs within the rs835486-rs835487-rs835488 region is the causal SNP, site directed mutagenesis was used to introduce the OA-associated allele of each SNP individually into the non-OA AAC haplotype. Introduction of the OA-associated G allele of rs835486 had no effect on enhancer activity of the rs835486-rs835487-rs835488 region in either cell line (Fig 2B and S3C Fig). However, there was significantly higher enhancer activity after introduction of the OA-associated G allele of rs835487 in both SW1353 cells (1.72 fold, *p* < 0.001; Fig 2B) and MDA-MB-231 cells (1.73 fold, *p* < 0.001; S3C Fig). Introduction of the OA-associated T allele of rs835488 to create the AAT haplotype of rs835486-rs835487-rs835488 also increased enhancer activity by 1.95 fold in SW1353 cells (*p* < 0.001, Fig 1B) and 2.24 fold in MDA-MB-231 cells (*p* < 0.001, S3C Fig) relative to the AAC haplotype created by the C allele of rs835488. This data suggests that both rs835487 and rs835488 contribute to the haplotype differences in enhancer activity of this region. This was confirmed by cloning all three SNPs within the rs835486-rs835487-rs835488 region separately. As expected, no allelic differences in activity were observed for the rs835486 SNP (Fig 2C). However, there was significantly increased activity of the OA risk alleles of both rs835487 and rs835488 relative to the non-OA allele of these SNPs in both cell lines (Fig 2C and S3D Fig). These two SNPs have a pairwise r^2^ of 0.962 in the European population and create two common haplotypes, AC and the OA-associated GT, which have a frequency of 0.633 and 0.358 respectively. Together, the luciferase data suggests that the OA region acts as a *cis*-acting regulator of gene expression, with the OA susceptibility residing at this locus potentially caused by the combined effects of allelic differences in regulatory activity of the rs835487 and rs835488 SNPs.

### Differential transcription factor binding to rs835487 and rs835488 Alleles

**Fig 3:**
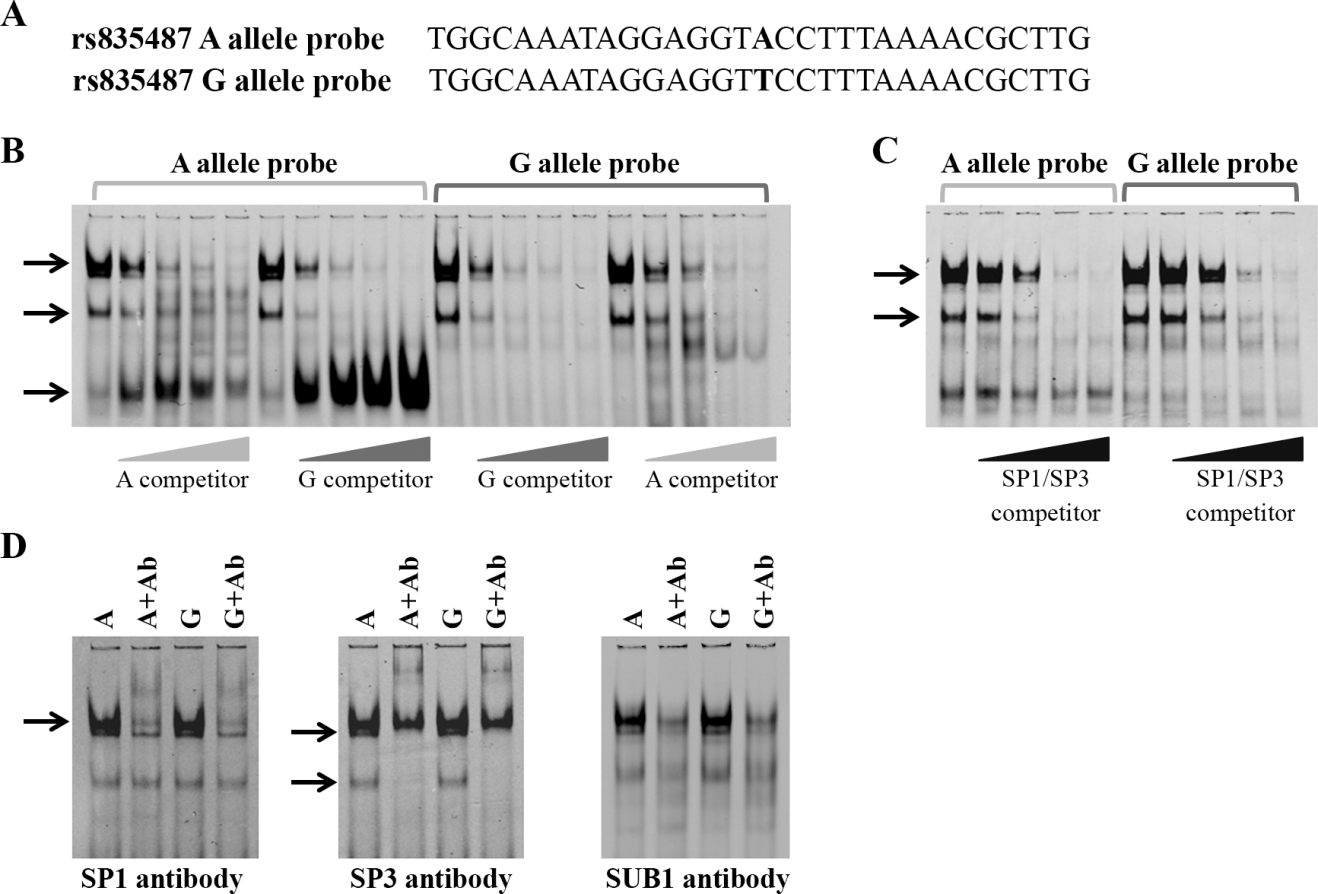
The OA-associated allele of rs835487 has increased binding of nuclear protein complexes containing SP1, SP3 and SUB1. (**A**) Positive strand sequence of the rs835487 double-stranded EMSA DNA probes with the alleles of the SNP highlighted in bold. (**B**) EMSA with rs835487 A and G allele probes with and without unlabelled A and G allele competitors. Unlabelled competitors were present in 0-, 10-, 25-, 50- and 100-fold molar excess as indicated and arrows denote rs835487-protein complexes. (**C**) EMSA using a competitor containing the SP1/SP3 consensus sequence. Arrows indicate the DNA-protein complexes that are reduced upon addition of the SP1/SP3 competitor. (**D**) Supershift of the rs835487-protein complexes with antibodies against SP1, SP3 and SUB1. Arrows denote the complexes that are supershifted with the antibody. Nuclear extracts from SW1353 human chondrosarcoma cell line were used in all EMSAs. Ab, antibody.

**Fig 4:**
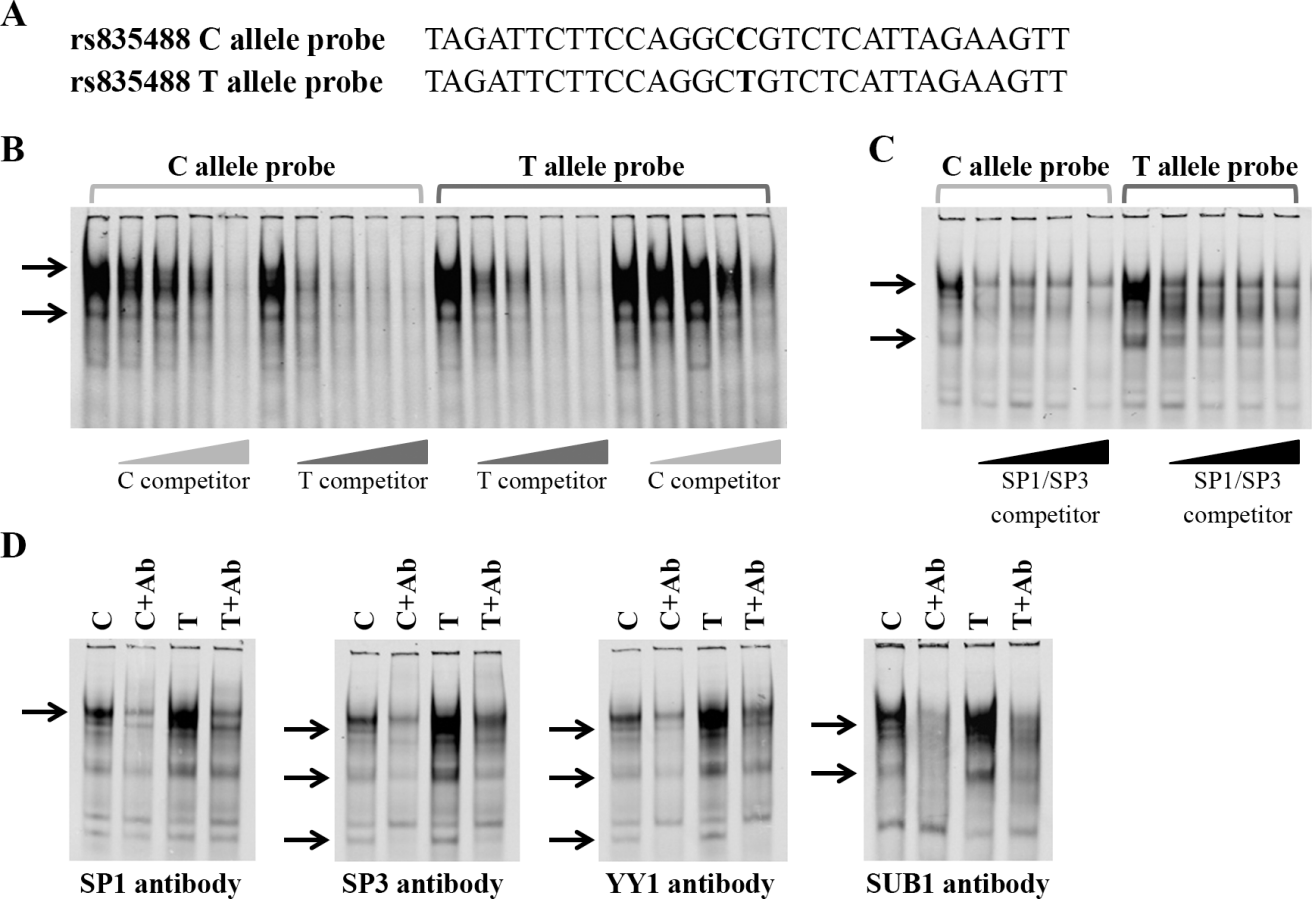
The OA-associated allele rs835488 has increased binding of nuclear protein complexes containing SP1, SP3, YY1 and SUB1. (**A**) Positive strand sequence of the rs835488 probes with the alleles of the SNP highlighted in bold. (**B**) rs835488 EMSA with the C and T allele probes with 0-, 10-, 25-, 50- and 100-fold molar excess of unlabelled C and T allele competitors; the rs835488-protein complexes are indicated by arrows. (**C**) rs835488 EMSA with SP1/SP3 consensus sequence competitors; DNA-protein complexes outcompeted by SP1/SP3 competitors are denoted by arrows. (**D**) Addition of antibodies against SP1, SP3, YY1 and SUB1 reduce formation of specific rs835488-protein complexes, as indicated by arrows. Nuclear extracts from SW1353 human chondrosarcoma cell line were used in all EMSAs. Ab, antibody.

The allelic differences in enhancer activity of rs835487 and rs835488 are likely to be caused by differences in protein binding and this was investigated using electrophoretic mobility shift assays (EMSAs). EMSAs were performed using nuclear extracts with double-stranded labelled DNA probes for both alleles of each SNP (Fig 3A, Fig 4A, S4A and S4C Fig).

Several protein complexes were found to bind to both alleles of rs835487 in SW1353 (Fig 3B, upper bands) and MDA-MB-231 (S4B Fig, upper bands) nuclear lysates, with an additional complex binding specifically to the A allele (Fig 3B and S4B Fig, lower band). For the upper complexes, protein binding was outcompeted at a lower concentration of unlabelled G allele competitor than the A allele competitor, indicating that these proteins bind more avidly to the OA risk G allele. Using the online databases matInspector, Promo3.0, TESS and TFSEARCH, we identified several transcription factors that were predicted to bind over rs835487 (S3 Table). Competition assays with competitors containing the consensus-binding site of these proteins and supershift assays using antibodies against these proteins were then performed (Fig 3C and 3D, and S5A Fig). Addition of SP1/SP3 competitors containing the consensus sequence GGGGGCGGGGG (Fig 3C) reduced the upper three protein-probe complexes in a concentration dependent manner, suggesting that SP1 and/or SP3 is a component of these complexes. None of the transcription factor consensus sequences outcompeted binding of the A-specific protein (data not shown). The upper rs835487-protein complex outcompeted with the SP1/SP3 consensus sequence was supershifted upon addition of an anti-SP1 antibody (Fig 3D, left panel), while the lower two complexes were supershifted with an anti-SP3 antibody (Fig 3D, middle panel), confirming that SP1 and SP3 bind over the rs835487 SNP *in vitro*. We have previously reported that the transcriptional co-activator protein SUB1 forms a complex with SP1 and SP3 [17] so we examined whether SUB1 also binds over the rs835487 SNP. Addition of an anti-SUB1 antibody reduced the formation of the SP1 containing complex and the upper SP3 containing complex (Fig 3D, right panel), but had little or no effect on the lower SP3-containing complex, suggesting that SUB1 stabilises the upper two complexes but not the smallest SP3 complex. Experiments with antibodies against other transcription factors did not supershift any of the rs835487 complexes, implying that it was not the presence of an antibody *per se* that created a shift but the presence of antibodies targeting SP1, SP3 and SUB1 (S5A Fig).

Several protein complexes were identified that bind to both alleles of the rs835488 SNP (Fig 4B and S4D Fig). These complexes bind more avidly to the OA risk T allele, with a lower concentration of the unlabelled C and T allele competitors required to outcompete the C allele complexes than the T allele complexes. Several transcription factors were predicted to bind to rs835488 (S3 Table) but only competitors for and antibodies against SP1 and SP3 reduced formation of the rs835488-protein complexes (Fig 4C and 4D). Addition of an anti-SP1 antibody reduced the formation of the largest rs835488-protein complex (Fig 4D, far left panel) and the formation of three complexes were reduced upon addition of an anti-SP3 antibody (Fig 4D, centre left panel). The region surrounding rs835488 (AGGCC/TGTCTC) is similar to the YY1 consensus sequence VDCCATNWY [27], and this transcription factor is known to form transcription complexes with both SP1 and SP3. We therefore investigated whether YY1 binds over rs835488. Addition of an anti-YY1 antibody reduced the formation of all three SP3-containing complexes (Fig 4D, centre right panel), suggesting that these complexes contain both SP3 and YY1. Formation of the SP1 and SP3-YY1 complexes was reduced in the presence of an antibody against SUB1 (Fig 4D, far right panel), indicating that SUB1 stabilises the formation of the rs835488 protein complexes. As for rs835487, shifts were not observed when antibodies targeting other *trans*-acting factors were used (S5B Fig).

In summary, the EMSA data highlighted allelic differences in protein binding to both rs835487 and rs835488 and identify SP1, SP3, YY1 or SUB1 as transcription factors differentially binding to this OA susceptibility locus.

### *CHST11* as a Potential Target for the OA Susceptibility

The above data suggests (but does not definitively prove) that 1) the OA association region acts as a distal regulator of gene expression, and 2) that the susceptibility mediated by this region functions through causing allelic differences in target gene expression due to differential protein binding to the rs835487 and rs835488 SNPs. The majority of enhancer-promoter interactions occur between elements located on the same chromosome (*in cis* interactions) rather than between elements on different chromosomes (*in trans* interactions). Studies of chromatin interactions in human cells using high resolution chromosome conformation capture technologies have demonstrated that most *in cis* interactions occur between elements within the same topological domain [28]. These topological domains have a median size of 1 Mb in humans, and the topological domain containing the rs835487-rs835488 region is located between chromosome 12:104,120,000–105,120,000 in the H1 human ES cell line and between chromosome 12:104,320,000–105,240,000 in the human IMR90 fibroblast cell line (http://chromosome.sdsc.edu/mouse/hi-c/download.html; [28]). To discover the putative target gene of the rs835487-rs835488 enhancer region, we concentrated on genes whose transcription start sites (TSS) were located within the rs835487-rs835488 topological domain. As the borders of this topological domain vary between cell types, we analysed the region located between 1 Mb up and downstream of the OA susceptibility region, that is the region on chromosome 12 between 104,059,920 and 106,062,650. This region contained the TSS of 18 genes and 1 pseudogene, which are listed in S4 Table. A literature search of these 18 genes identified *CHST11* as the most likely candidate gene of the rs835487-rs835488 enhancer based on the following observations: A) the orthologous region in rodents is part of a super-enhancer that regulates *Chst11* expression in proliferating and prehypertrophic chondrocytes ([15,16]; S2 Fig); B) according to the HaploReg V4 database, rs835487 acts as a *CHST11* expression quantitative trait locus (eQTL), albeit in cerebellum and temporal cortex [29,30]; C) mice lacking CHST11 protein have severe chondrodysplasia, indicating *Chst11* is required for normal skeletogenesis [31]; D) CHST11 sulfates chondroitin to produce chondroitin sulfate, a key component of cartilage proteoglycan that is crucial for extracellular matrix interactions with growth factors and other signalling molecules [7]; E) *CHST11* is differentially methylated in OA cartilage relative to non-OA cartilage [32]; and F) *CHST11* expression is upregulated in cartilage from patients with OA [33]. We therefore focussed on this gene.

**Fig 5:**
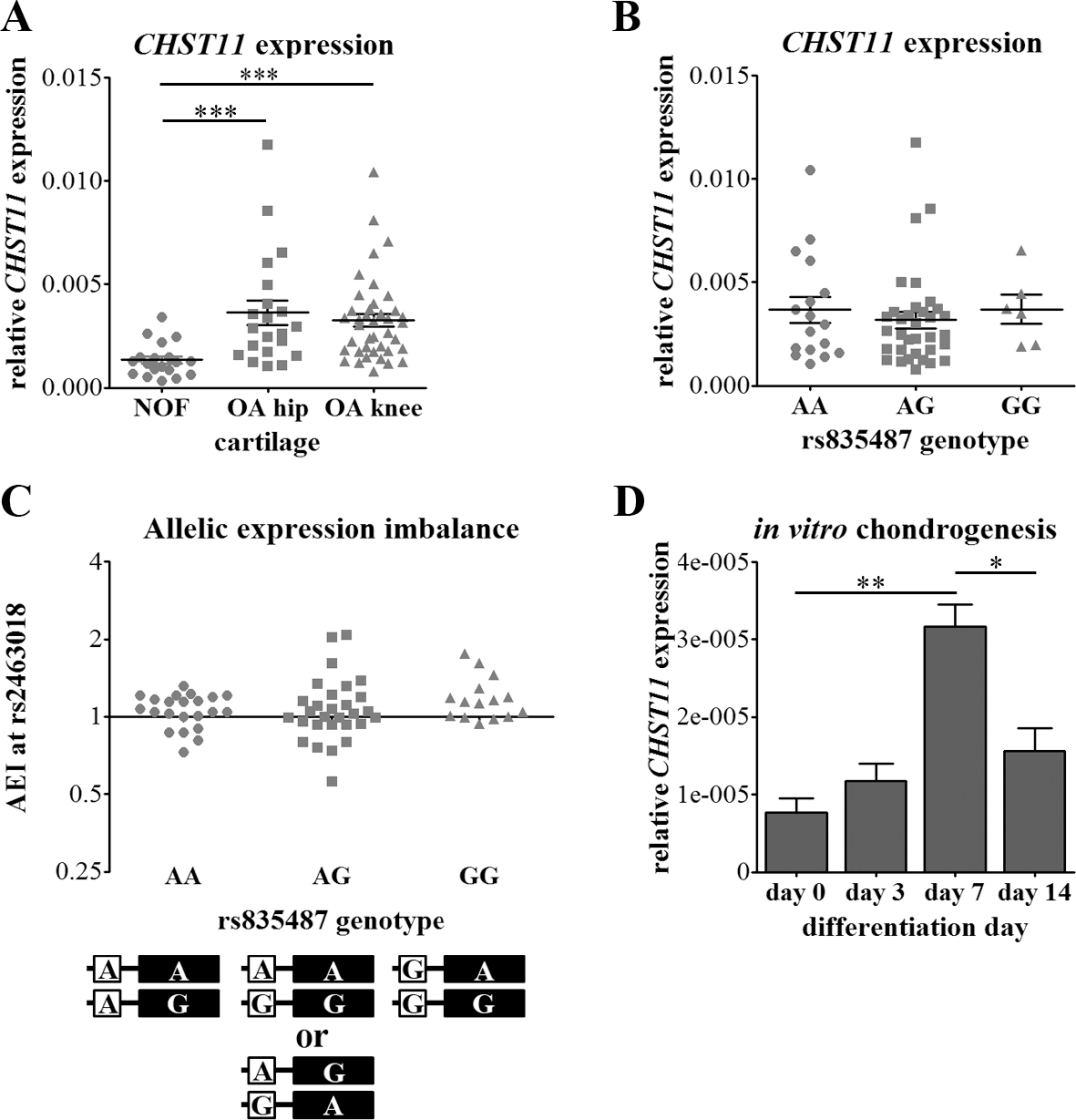
*CHST11* expression in adult cartilage and during chondrogenesis. (**A**) *CHST11* expression in cartilage samples from neck of femur (NOF) fracture patients, OA hip and OA knee patients. (**B**) *CHST11* expression in OA hip and OA knee cartilage stratified by genotype at the OA SNP rs835487. (**C**) Allelic expression imbalance (AEI) of the *CHST11* transcript SNP rs2463018 stratified by genotype at rs835487 in NOF, OA hip and OA knee cartilage. Although only rs835487 genotypes are shown, rs835487 and rs835488 had an r^2^ of 1 in these 66 individuals, with the AA, AG and GG genotypes at rs835487 being in complete LD with the CC, CT and TT genotypes at rs835488 respectively; rs835487 and rs835488 genotypes are therefore directly interchangeable in this data set. rs2463018 mRNA allelic ratios were normalised to cartilage DNA from the same individual. The schematic diagram below the chart indicates the different haplotype combinations of rs835487 (open box) and rs2463018 (black rectangle). For (**A**), (**B**) and (**C**), each circle, square and triangle represents data from a single individual. (**D**) Expression of *CHST11* during *in vitro* chondrogenic differentiation of MSCs (3 donors). For (**A**), (**B**) and (**D**), *CHST11* expression was normalised to the housekeepers *18s*, *GAPDH* and *HPRT1*, and the mean ± standard error is indicated by the error bars. *p < 0.05, **p < 0.01, ***p < 0.001, Mann-Whitney *U* test. The data points that enabled construction of panel D of this figure can be found in S5 Table.

We measured the expression of *CHST11* in cartilage from patients who had undergone joint replacement surgery either due to primary hip OA (n = 22) or primary knee OA (n = 40), or due to neck of femur (NOF) fracture (n = 19). *CHST11* was upregulated approximately 2.7 fold in OA hip and OA knee cartilage relative to non-OA hip cartilage from the NOF individuals (*p* < 0.001, Fig 5A).

Given our hypothesis that the rs835487-rs835488 region acts as an enhancer, and that there are allelic differences in enhancer activity *in vitro*, we stratified our OA hip and OA knee cartilage *CHST11* expression data by genotype at rs835487 to determine if the rs835487-rs835488 region acts as an eQTL on this gene. We observed no correlation between rs835487 genotype and overall *CHST11* expression in cartilage when OA samples were combined (Fig 5B) or, given that the association marked by rs835487 is specific to hip OA, when the data was stratified by joint site (S6A–S6C Fig). The effect of rs835487 genotype on *CHST11* expression may be overshadowed or even hidden by the large inter-individual variability in expression levels, especially as the so far characterised OA SNP eQTLs have small, subtle effects on gene expression [9–11]. We therefore investigated potential effects of rs835487 and rs835488 genotype on *CHST11* expression further using allelic expression imbalance (AEI) analysis; this technique measures the transcriptional output of each allele of a SNP in heterozygous individuals and therefore is not affected by variability in gene expression between individuals. As the OA susceptibility SNPs are intronic, we used the *CHST11* 3'UTR transcript SNP rs2463018 as a proxy; although this SNP has an r^2^ < 0.001 with rs835487, it has a heterozygous frequency of 41%, meaning that a large proportion of individuals would be eligible for analysis. We analysed AEI in cartilage from 66 individuals heterozygous for the rs2463018 transcript SNP. Our genotyping of these samples revealed that rs835487 and rs835488 were in perfect LD (r^2^ = 1), with individuals with the AA, AG and GG genotypes at rs835487 having the CC, CT and TT genotypes at rs835488 respectively. Twenty-two of the 66 individuals were homozygous for the non-OA A allele of rs835487, 29 were heterozygous for rs835487 and 15 were homozygous for the OA-associated G allele of rs835487. Although AEI was observed at rs2463018 in cartilage, the level and direction of AEI did not correlate with genotype at rs835487 (and therefore not at rs835488 either) (Fig 5C). Given that the rs835487 OA association is specific to hip OA, we further stratified the data based on cartilage joint of origin (S6D–S6F Fig), but found no evidence that rs2463018 AEI is driven by rs835487 or rs835488 genotype.

Our expression data does not support the OA association signal as acting as an eQTL on *CHST11* in the adult cartilage that we investigated. In mice, *Chst11* is expressed in growth plate chondrocytes, where the protein is required for normal growth plate patterning [31]. To assess if *CHST11* is also expressed in human chondrocytes during chondrogenesis, we *in vitro* differentiated human iliac crest derived mesenchymal stem cells (MSCs) using the Transwell chondrogenic differentiation model [34,35]. *CHST11* was expressed in undifferentiated (day 0) MSCs, and was upregulated approximately three-fold during the first 7 days of chondrogenesis (*p* = 0.0021; Fig 5D), when the cells are proliferating. *CHST11* levels fell 2-fold between day 7 and day 14 (*p* = 0.0126) by which time the chondrocytes have stopped proliferating and express the hypertrophic marker *COL10A1* [34,35].

## Discussion

Our analysis revealed that rs835487 and rs835488 are the most likely candidate SNPs for the OA susceptibility that resides at chromosome 12q23 and within intron 2 of *CHST11*, with their disease associated GT haplotype having significantly different *in vitro* enhancer activity relative to the AC haplotype. However, when we examined the expression of *CHST11* in the cartilage of OA patients, we did not detect a correlation between expression and genotype, even when using the sensitive AEI approach. However, due to the absence of *CHST11* transcript SNPs in high LD with rs835487, we had to use a transcript SNP not in LD with rs835487 for AEI, limiting the resolution of the assay. Given that most regulatory SNPs have only subtle effects on their target gene expression, we cannot rule out the possibility that other SNPs in higher LD to the transcript SNP may have masked the effects of an rs835487 AEI on *CHST11* expression in this assay. Nevertheless, we found no evidence that rs835487 acts as a *CHST11* cartilage eQTL and one explanation for this could be that *CHST11* is not the target of the OA susceptibility. This is of course perfectly possible, although the orthologous enhancer regions in mice and rats are part of a super-enhancer that regulates *Chst11* in proliferating and prehypertrophic chondrocytes [15,16] and rs835487 is associated with a *CHST11* eQTL in cerebellum and temporal cortex [29,30]. It may be therefore that, like many enhancers, the gene regulatory activity of the OA LD region is temporally and spatially restricted rather than being active throughout the life-course and would not therefore have been detectable in our analysis of cartilage from elderly patients.

*CHST11* is a very compelling functional candidate based on the known role of its encoded protein, carbohydrate sulfotransferase 11, on cartilage extracellular matrix biosynthesis. This enzyme sulfates the glycosaminoglycan (GAG) chondroitin sulfate (CS) [7]. GAGs are chains of disaccharides that bind to a core protein to form proteoglycan [36]. Alongside collagens, proteoglycans are the most abundant components of the cartilage extracellular matrix (ECM), with the principal cartilage proteoglycan being aggrecan. The high sulfate content adds negative charge to the proteoglycan, which contributes to the water binding capacity of the molecule. In the case of aggrecan and cartilage, this leads to the swelling observed in the healthy tissue that enables the cartilage to resist load. Sulfation also mediates interaction of the proteoglycan with extracellular molecules, including growth factors and cytokines [36]. Aberrant GAG sulfation can lead to a range of skeletal pathologies, including abnormal growth plate development, bone shortening and chondrodysplasia [37–39]. Inactivation of the mouse homolog of *CHST11* leads to an imbalance of chondroitin sulfation and loss of cartilage integrity [31].

If *CHST11* is the target of the OA susceptibility we therefore speculate that this reflects usage of the intron 2 enhancer during cartilage development rather than in the adult cartilage that we investigated. Many enhancers have been reported to act in a specific-cell type and at a specific developmental stage. For example, a conserved enhancer region within the *BCL11A* gene, which acts as a negative regulator of foetal haemoglobin levels, acts as an enhancer of this gene specifically in the erythroblast cells, and not in B lymphocytes, even though *BCL11A* is expressed 10-fold higher in B lymphocytes than erythroblasts [40]. Furthermore, the enhancer drives expression of *BCL11A* in fetal liver cells, the site of erythropoiesis, in a developmentally-specific pattern. Many enhancers that regulate chondrogenic gene expression during skeletogenesis are also temporally and spatially restricted, including those of *Sox9* [41] and *Acan* [42]. Several OA genetic susceptibility loci map near to or encompass genes that play important roles during skeletogenesis [5] and it has been hypothesised that OA loci do act during joint development by causing subtle alterations in joint architecture and/or cartilage/bone composition that predispose the individual to OA development later in life [43,44]. As already noted, *Chst11* is expressed in the developing limb during mouse embryonic skeletogenesis, including in proliferating and pre-hypertrophic but not hypertrophic growth plate chondrocytes [7,16,31]. Although it is not possible to look at *CHST11* expression during human skeletal development, we did observe dynamic expression of this gene during *in vitro* chondrogenesis of human MSCs, with *CHST11* expression peaking at the end of the proliferative phase. Therefore, we suggest that *CHST11* could be a further example of an OA genetic susceptibility that mediates its effect during development of the joint. To assess if this is the case, the enhancer marked by rs835487 and rs835488 could be deleted in MSCs by, for example, the use of CRISPR-Cas [45] and the effect that this has on chondrogenesis and *CHST11* expression directly assessed. If no effect on *CHST11* expression were observed but there was an effect on chondrogenesis, then a reasonable conclusion would be that *CHST11* is not the target and as such an expression analysis of all other genes from within the rs835487-rs835488 topological domain would be merited, in both the deletion model and patient cartilage.

## Conclusions

The OA susceptibility residing within *CHST11* is mediated by differential protein binding to the alleles of rs835487 and rs835488, which are located within a region that functions as an enhancer of transcription. SP1, SP3, YY1 and SUB1 are the transcription factors whose binding to the enhancer are altered by the OA associated SNPs.

## Supporting Information

S1 Fig ClustalW alignments of the SNPs within the enhancer region that have an r^2^ of 0.8 or above with the rs835487 SNP that marks the OA association at this locus. In mammalian species that have homologous regions to the human LD block within the orthologous *CHST11* gene, the 60 bp regions surrounding each of the seven SNPs within the OA signal (rs835486, rs835487, rs835488, rs835490, rs835491, rs835492 and rs835493) were aligned using ClustalW. Each SNP is highlighted in yellow. Primate sequences are in black, Cetartiodactyla in blue, Prissodactyla in red, Carnivora in green, Chiroptera in orange, and Afrotheria in purple. rs835486 is only present in primates, with the A allele being ancestral. The region containing rs835487 is conserved in the largest number of mammalian species (n = 25). The rs835488 region is conserved in the second largest number of mammalian species (n = 21), with the OA–associated T allele appearing to be the ancestral allele. In the primate lineage, there are insertions upstream of rs835490 and rs835491, which may potentially introduce or delete transcription factor binding sites that are conserved in the other mammalian species.
(PDF)

S2 Fig The LD region shares homology with an enhancer that regulates *Chst11* expression in rat and mouse proliferating chondrocytes. (**A**) UCSC genome browser view of the LD block and surrounding region. The LD block was defined as the region containing SNPs with r^2^ of 0.8 or greater with the OA SNP rs835487 within the EUR population and is indicated by the red rectangle. The black rectangles indicate the regions with homology to the SOX9 and SOX5/6 ChIPseq peaks present in rat proliferating/early prehypertrophic growth plate chondrocytes (RCS tracks; Liu and Lefebvre 2015 [16]), the SOX9 ChIPseq peaks in proliferative and prehypertrophic rib chondrocytes from postnatal day 1 mice (Mm P1 rib SOX9; [15]), and the H3K4me1 and H3K27Ac ChIPseq peaks from E14.5 mouse limb (Mm limb H3K4me and Mm limb H3K27Ac respectively). Homologous regions were identified using the UCSC LiftOver tool between the rat RGSC 5.0/rn5, the mouse NCBI37/mm9 and the human GRCh37/hg19 assemblies. The filled black rectangles within the homologous blocks represent the regions with over 70% identity between the human and rat/mouse nucleotide sequences; these regions were identified using the blastn sequence alignment tool that is optimised for ‘somewhat similar sequences’. The chromatin state segmentation tracks for human bone-marrow derived mesenchymal stem cells (Hs BM-MSC), *in vitro* differentiated chondrocytes (Hs Chondrocytes) and primary osteoblasts (Hs osteoblasts) from the RoadMap Epigenome project, human transcription factor binding sites (Hs TF ChIP-seq) and DnaseI hypersensitivity sites (Hs DHS clusters) from the ENCODE project, and human enhancer transcripts from the FANTOM 5 consortium (Hs transcribed enhancer; [26]) are also indicated. The seven vertical lines mark the positions of, from left to right, rs835486, rs835487, rs835488, rs835490, rs835491, rs835492 and rs835493 (OA SNPs). (B) Alignment of the LD region in 29 mammalian species generated using the Multiz tool in UCSC.
(PDF)

S3 Fig The OA risk locus has haplotype-specific differences in luciferase reporter activity. (**A**) to (**D**) Luciferase enhancer assays in the MDA-MB-231 human adenocarcinoma cell line using the pGL3 promoter plasmids. The vectors were co-transfected with the pRL-TK Renilla plasmid, and luciferase/renilla values normalised to the empty pGL3-enhancer vector. (**A**) Luciferase activity of the AAC and OA-associated GGT haplotype of the region containing the rs835486, rs835487 and rs835488 SNPs. (**B**) Luciferase activity of the CCAC and OA risk TTGT haplotype of the region containing rs835490, rs835491, rs835492 and rs835493. (**C**) Luciferase activity of the rs835486-rs835487-rs835488 vectors containing the AAC, GAC, AGC and AAT haplotypes. Mutating the non-OA allele into the OA-risk allele of rs835486, rs835487 or rs835488, respectively, created the GAC, AGC and AAT vectors. (**D**) Luciferase activity of the two alleles of rs835487 only (left) or rs835488 only (right). There is increased luciferase activity of the OA associated alleles of both SNPs. Data shown is the mean ± standard error of at least four independent experiments, each with five technical repeats. *p < 0.05, ***p < 0.001, Mann-Whitney *U* test. N/S, not significant. The data points that enabled construction of this figure can be found in S5 Table.
(PDF)

S4 Fig rs835487 and rs835488 competiton EMSAs with MDA-MB-231 protein lysates. (**A**) Positive strand sequence of the rs835487 double-stranded EMSA DNA probes with the alleles of the SNP highlighted in bold. (**B**) MDA-MB-231 nuclear lysate EMSA with rs835487 A and G allele probes with and without unlabelled A and G allele competitors. Unlabelled competitors were present in 0-, 10-, 25-, 50- and 100-fold molar excess as indicated and arrows denote rs835487-protein complexes. (**C**) Positive strand sequence of the rs835488 probes with the alleles of the SNP highlighted in bold. (**D**) rs835488 EMSA with the C and T allele probes with 0-, 10-, 25-, 50- and 100-fold molar excess of unlabelled C and T allele competitors and MDA-MB-231 nuclear proteins.
(PDF)

S5 Fig Supershift assays for the rs835487 and rs835488 protein complexes. (**A**) rs835487 A allele gel supershift assays with antibodies against 17 transcription factors. The rs835487-protein complexes are only supershifted upon addition of antibodies against SP1 or SP3, with no supershifts observed for the other 15 antibodies tested. The SP1-containing complexes are indicated by the long arrows, with the short arrow indicating SP3-containing complexes. (**B**) rs835488 C allele supershift assays with antibodies against 16 transcription factors. Addition of an anti-SP1 antibody reduces formation one of the rs835488-protein complexes (long arrow), and formation of two complexes are decreased upon the addition of anti-SP3 or anti-YY1 antibodies (short arrows). An anti-SUB1 antibody reduces formation of all three SP1/SP3/YY1 containing complexes. Nuclear extracts from SW1353 human chondrosarcoma cell line were used in all EMSAs. Ab, antibody. Ctl Ab, anti-PAX6 polyclonal antibody used as an IgG control.
(PDF)

S6 Fig Association analysis of *CHST11* expression with rs835487 genotype. (**A**) to (**C**) *CHST11* expression in (**A**) neck of femur (NOF) fracture, (**B**) OA hip and (**C**) OA knee cartilage stratified by genotype at the OA-associated SNP rs835487. Expression was measured by qRT-PCR and normalised to the housekeeping genes *18s*, *HPRT1* and *GAPDH*. The error bars indicate the mean ± standard error. Due to the low number of GG homozygotes in the OA hip analysis, the heterozygotes and GG homozygotes were combined as G carriers and compared to the AA homozygotes. (**D**) to (**F**) Allelic expression imbalance (AEI) of the *CHST11* transcript SNP rs2463018 stratified by genotype at the OA SNP rs835487 in (**D**) NOF, (**E**) OA hip and (**F**) OA knee cartilage. Although only rs835487 genotypes are shown, rs835487 and rs835488 had an r^2^ of 1 in these 66 individuals, with the AA, AG and GG genotypes at rs835487 being in complete LD with the CC, CT and TT genotypes at rs835488 respectively; rs835487 and rs835488 genotypes are therefore directly interchangeable in this data set. Allelic ratios were normalised to cartilage DNA from the same individual. For (**A**) to (**F**), each circle, square and triangle represents data from a single individual.
(PDF)

S1 Table A list of primers used in this study. The bold and underlined nucleotides in the Site directed mutagenesis primers are the mutagenized SNP alleles; the bold and underlined nucleotides in the EMSA probes are the SNP sites; the underlined sequences in the SP1/SP3 primers is the consensus sequence for the transcription factors.
(PDF)

S2 Table Conservation of the OA-associated enhancer region in mammals. The OA associated region maps to chromosome 12 within intron 2 of the *CHST11* gene at hg19:105,059,911–105,062,613 and contains seven SNPs in high LD (r^2^ > 0.8). To identify homologous enhancer regions in other mammalian species, the 2.7 kb genomic region encompassing the human OA locus was aligned against the genomes of other mammals using the UCSC BLAT software. The homologous sequences from species where the enhancer was conserved within intron 2 of *CHST11* were aligned using ClustalW software. The OA enhancer region is present in eutherian mammals, but not in monotremes or marsupials. The enhancer region has diverged in the Rodentia and Lagomorpha lineages, with some regions being absent. The allele present in the reference genome of the different species at each SNP is listed in the table. For humans, the common non-risk allele is given followed by the minor OA risk allele. **Bp del**; for rs835487, the region surrounding the SNP is conserved in elephants although the SNP nucleotide itself has been deleted. **C del**; for rs835490, the region 5ʹ to the SNP has either been lost from the Superorder Laurasiatheria or gained in the primate order. **4bp in**; there has been a 4 bp TCAG insertion 2 bp upstream of the rs835491 SNP in the New World primates (in bold) and Squirrel Monkey genomes and a 4 bp GAAT insertion 5 bp upstream of rs835492 in the Lemur genome. **15bp in**; there is a 15 bp insertion 2 bp 5ʹ to the rs835491 SNP in Marmosets.
(PDF)

S3 Table Transcription factors identified using the online databases matInspector, Promo3.0, TESS and TFSEARCH as predicted to bind over rs835487 and rs835488. The consensus and competitor sequences used in the subsequent competition EMSAs are also listed.
(PDF)

S4 Table Genes with transcription start sites located within the 2Mb region surrounding rs835487. Details of the gene transcription start site (TSS) and function of the gene/encoded protein are listed, together with information on whether they demonstrate altered expression in OA cartilage versus non-OA cartilage (using data from [32]), or whether they show altered expression in OA cartilage versus non-OA cartilage (using data from [22,33]). Also listed is whether they have a reported role in the musculoskeletal system based on searches of PubMed (http://www.ncbi.nlm.nih.gov/pubmed) and OMIM (http://www.ncbi.nlm.nih.gov/omim) and information from mice deletion models.
(PDF)

S5 Table The data points used for the construction of Fig 2, S3 Fig and Fig 5D. The first sheet contains the data points for Fig 2 and the luciferase assay of SW1353 human chondrosarcoma cells. The second sheet contains the data points for S3 Fig and the luciferase assay of MDA-MB-231 human adenocarcinoma cells. The third sheet contains the data points for Fig 5D and MSC chondrogenesis.
(XLSX)
