# Supplementary material for: Functional Characterization of the Osteoarthritis Susceptibility Mapping to CHST11—A Bioinformatics and Molecular Study
Source: PLoS One. 2016 Jul 8;11(7):e0159024. doi: 10.1371/journal.pone.0159024 (PMC4938163; doi:10.1371/journal.pone.0159024)
Supplement: S1 Fig — In mammalian species that have homologous regions to the human LD block within the orthologous CHST11 gene, the 60 bp regions surrounding each of the seven SNPs within the OA signal (rs835486, rs835487, rs835488, rs835490, rs835491, rs835492 and rs835493) were aligned using ClustalW. Each SNP is highlighted in yellow. Primate sequences are in black, Cetartiodactyla in blue, Prissodactyla in red, Carnivora in green, Chiroptera in orange, and Afrotheria in purple. rs835486 is only present in primates, with the A allele being ancestral. The region containing rs835487 is conserved in the largest number of mammalian species (n = 25). The rs835488 region is conserved in the second largest number of mammalian species (n = 21), with the OA–associated T allele appearing to be the ancestral allele. In the primate lineage, there are insertions upstream of rs835490 and rs835491, which may potentially introduce or delete transcription factor binding sites that are conserved in the other mammalian species. (PDF) [file pone.0159024.s001.pdf]

rs835486 alignment - present in squirrel monkey and old world monkeys only

```
human              TCTCTTTTAATCTTATTTAGACTATCATGTATAGAATTAAGATTGAATACTTTATTTAAAT
chimp              TCTCTTTTAATCTTATTTAGACTATCATGTATAGAATTAAGATTGAATACTTTATTTAAAT
gorilla            TCTCTTTTAATCTTATTTAGACTATCATGTATAGAATTAAGATTGAATACTTTATTTAAAT
orang-utan         TCTCTTTTAATCTTATTTAGACTATCATGTATAGAATTAAGATTGAATACTTTATTTAAAT
gibbon             TCTCTTTTAATCTTATTTAGACTATCATGTATAGACTTGAGATTGAATACTTTATTTAAAT
rhesus monkey      TCTCTTTTAATGATATTCAGACTATCATGTATAGAATTGAGATTGAGTACTTTATTTAAAT
baboon             TCTCTTTTAATGATATTCAGACTATCATGTATAGAATTGAGATTGAGTACTTTATTTAAAT
squirrel monkey    TCTCTTTTAATCATATTTAGACTAACATGTATAGAATTGGGATTGAACACTTT--------
                   *********** :**** ******:**********.**..******. *****
```

rs835487 alignment

```
human              GTATGGCATGGGGCCTGGCAAATAGGAGGTACCTTTAAAACGCTTGTTGAGCAGAACATTC
chimp              GTATGGCATGGGGCCTGGCAAATAGGAGGTGCCTTTAAAACGCTTGTTGAGCAGAACATTC
gorilla            GTATGGCATGGGGCCTGGCAAATAGGAGGTGCCTTTAAAACGCTTGTTGAGCAGAACATTC
orang-utan         GTATGGCATGGGGCCTGGCAAATAGTAGGTGCCTTTAAAACACTTGTTGAGCAGAACATTC
gibbon             GTATGCCATGGGGCCTGGCAAATAGTAGGTGCCTTTAAAACACTTGTTGAGCAGAACATTC
rhesus monkey      GTATGGCATGGGGCCTGGCACATCGTAGGTACCTTTAAAACGCTTGTTGAGCAGAACATTC
baboon             GTATGGCATGGGGCCTGGCAAATCGTAGGTACCTTTAAAACGCTTGTTGAGCAGAACATTC
squirrel monkey    GTATGGCATGGGGCCTCACAAATAGTAGGTGCCTTTAAAACACTTGTGGAGTGGAACATTC
marmoset           GTATAGTCCGGGGCCTGGCAAATAGTAGGTGCGTTTAAAACACTTGTGGAGTGGAACATTC
bushbaby           GTATGGTGTGGACCCTGGCAAAGAGTAGGTACCTTAAAAATGCTTGTTAAGTGGAACGTTC
lemur              ATGTGGCAGGGGACCTGGCAAATAGTAGATGCCTTAAAAATGCTTATTGAGTGGAACATTC
pig                CCTATTCCTTCTGCCCAGCAAACAGTAGGTGCCTTAAAAATACTTGCTGTATGAACCATGT
alpaca             ------------GCCTGGCAAATAATAGGTTCCTTAAAAACACTTGTTAAATGAAACACTC
minke whale        CATATTCCTCCTGCCTGGCAAATAGTAGGTGCCTTAAAAATACTTGTTGAATGAAACATTC
dolphin            CATATTCCTCCTGCCTGGCAAATAGTAGGTGCCTTAAAAATACTTGTTGAATGAAACATTC
sheep              TATATTCTTCTTGCTTGGCAAATTGTAGATGCCTTGCAAATACTTGCTGAATGAAACATTC
cow                TATATTCCTCTTGCTTGGCAAATAGTAGATGCCCTGAAAATACTTGCTGAATGAAACATTC
horse              GGCTGGCATGGGGCCCAGCAAAGAGGAGATGCCTTCAAAGTGCTGGTTGCATGAAACATGC
white rhino        GTCTGGCATGGGGCCCGGCAAAGAGGAGATGTCTTAAAAGTGCTTGTTGAATGAGACATTC
cat                GTCTGGAATGAGACCTGGCAAATAAAAGGCGCCTTAAAAATGCT-GTTGAATAAAATGCTC
ferret             GGCCGGAATGGCACGTGGCAAATAAAGGGTGCCTTTAAAATGCTCGTTGAATAAAGTTT--
dog                GTCCAGCATGGGACCTGGCAAATAAAAGGTGTCTTCAAAA-GCTTATTGAATAAAATTT--
panda              GGCTGGCATGGCACCTGGCAAATAAGAGGTGCCTTAAAAATGCTTGTTGAATAAAATTT--
elephant           ATCTGGCATAGGGCCTGGCAAATGATAGGT-CCCTAAAAATGCTTGTTGAATGGAACATTT
manatee            ATCTTGGACAGGGCCTGGCAAATGATAGGTGCCTAAA-AATGCTTGTTGAATGGAACATTT
                                *   .**.*  . .*.     : . *. .** .  . . ...
```

rs835488 alignment

```
human              TGACTGCAAACTTTTAAGATTCTTCCAGGCCGTCTCATTAGAAGTTGACAGCACTTTGGCA
chimp              TGACTGCAAACTTTTAAGATTCTTCCAGGCTGTCTCATTAGAAGTTGACAGCACTTTGGCA
gorilla            TGACTGCAAACTTTTAAGATTCTTCCAGGCTGTCTCATTAGAAGTTGACAGCACTTTGGCA
orang-utan         TGACTGCAAACTTTTAAGGTTATTCCAGGCTGTCTCATTAGAAGTTGACAGTACTTCGGCA
gibbon             TGACTGCAAACTTTTAAGGTTCTTCCAGGCTGTCTCATTAGAAGTTGACAGCACTTTGGCA
rhesus monkey      TGACTACAAACTTTTAAGGTTCTTCCAGGCTCTCTCATTAGAAGTTGACAGCACTTTGGCA
baboon             TGACTACAAACTTTTAAGGTTCTTCCAGGCTCTCTCATTAGAAGTTGACAGCACTTTGGCA
marmoset           TGACTGCAAACATTTAAGGTTCTTCCAGGTTCTTTCATTAGAATTTGACAGCATTTTGGCA
squirrel monkey    TGATTGCAAACATTTAAGGTTCTTCCAGGTTCTCTCGTTAGAAGTTGACAGCGTTTTGGCC
bushbaby           GGACTGCAGACATT-AAGATTCTTCCAAGTTCTCCCGT---AAGTTGACAGCATCTTGGTG
pig                TGATGGCAAACAT-TAAGGCTCTTCCAGCCGCTCTCCTATGATGGTCACAGCATCTTGGTG
alpaca             ------CTAACAT-TAAGGTTCTTCTGGCCTCTCTCATAAGATGTTGACAGCATCTTGGTG
minke whale        TGACTGCAAACGT-TAAGGTTCTTCTAGCCCCTCTCATAAAATGTTGACAGCATCTTGGTG
sheep              TGGCTGCAAACAGTTAAGATTCTTTTAGCCTCTTTCATAAGATGTGGATCGCTTCTTGGTG
cow                TGGCTGCAAACAG-TAAGTTTCTTTTAGCCTCTCTCATAG-ATGTGGATAGCATCTTGGTG
horse              TGACCGTAAACAT-TCGAGCTCTTCCAGCCTCCCTCCTGAGACGGTGACAGCATCTTCGTG
white rhino        TGACCGTAAACAG-TAAGGTTCTTCTAGCCTCCCTCATGAGACGTTGACAGCATCTTCGTG
ferret             TGACTGCCGGCA---------------GCCTGTCTCATGAGAGGTGGGCAGTATCTCGGCG
dog                TGACCATAGGTGG---------------CCTTTCTCGTAAGAGGTTGACAGCATCTTGGTG
panda              TGACTGTAGGCAG---------------CGTCTCTCGTAAGAGCTCGACAGCATCTGGGTG
megabat            ------CAAGCGT-TAAGATTCTTCTAGCCTCT--CTTAAGAGGTTGACAGTATATCAGTG
                           ..                         * *   *     . .*    *  *
```

```
rs835490 alignment
human             CGTCC-CCTAGTTCCGACGTTTATTATCCCCCCGGGTGGATAGCACTAATTCATACCAGTC
chimp             TGTCC-CCTAGTTCCGACGTTTATTATCCCCCTGGGTGGATAGCACTAATTCCTACCAGTC
gorilla           CGTCC-CCTAGTTCCGACGTTTATTATCCCCCCAGATGGATAGCACTAATTCATACCAGTC
orang-utan        CGTCC-CCTAGTTCCGACGTTTATTATCCCCCCAGGTGGATAGCACTAATTCATACCAGTC
gibbon            CGTCC-CCTAGTTCCGACATTTATTAT-CCCCCGGATGGATAGCACTAATTCATACCAGTC
rhesus monkey     CGTCC-CCTAGTTCCGACATTTATTATCCCCCCGGGTGGATAGCACTAATTCATACCAGTC
baboon            CGTCC-CCTAGTTCCGACATTTATTATCCCCCCGGGTGGATAGCACTAATTCATACCAGTC
squirrel monkey   CATCC CCTGGTTCTGACGTTTATTATCCCCCCGGGTGGATAGCACTAGTTCATACCAGTC
marmoset          CATCC-CCTGGTTCTGACGTTTATTATCCCCCCGGGTGGGTAGCACTAGTTCATACCAGAC
lemur             CGCCCGCCTGGTTCTGACCCTTGATATCTCCTGGGGTGGACAGAACCGATTCTTACCAGTC
bushbaby          CGACT-------TCCGATACT---TACCACCCAGGGTGGACAGCACCAATTCATACCAGTC
pig               TGCGTGC-----------------------CTGGGGTGGGCGGCTCTACTCCATACCCGTC
alpaca            TGTGTGC-----------------------CCAGGGTGGGAAGCATTAATTCATACCAGTC
minke whale       TGTGCGC-----------------------CTGGGGTGGTCGGCAGTAATTCGTACCAGTC
cow               TGTGTG------------------------CTGGGGTGGACAGCAGTAATTCATACCAGTC
sheep             TGTGCGC-----------------------CAGGGGTGGACAGCAGTAATTCCTACCAGTC
dog               TGCCCCC------------------CTCCCCTGGGGTGGACAGCACTAATTTATACTCGTC
panda             TGCCCCC----------------CGCCCCGCCCCGGTGGACAGCACTGATTTCTACTCATC
elephant          ------------------------------------TGGACAGCACTAATTCAGACCAGGG
                                                      ***  .*.:  . *    ** ..:


rs835491 alignment
human             ACTAATTCATACCAG-----------TCTGTCCGATGGCAGCAACCCCCATAGATGTATGGGT
chimp             ACTAATTCCTACCAG-----------TCTGTCCGATGGCAGCAACCCCCATAGATGTATGGGT
gorilla           ACTAATTCATACCAG-----------TCTGTCCGATGGCAGCAACCCCCATAGATGTATGGGT
orang-utan        ACTAATTCATACCAG-----------TCTGTCTGATGGCAGCAACCCCCTTAGATGTA-----
gibbon            ACTAATTCATACCAG-----------TCTGTCCAATGGCAGCGACCCCCATAGATGTATGGGT
rhesus monkey     ACTAATTCATACCAG-----------TCTGTCTGATGGCAGCAACCCCCATAGATGTATGGGT
baboon            ACTAATTCATACCAG-----------TCTGTCTGATGGCAGCAACCCCCATAGATGTATGGGT
squirrel monkey   ACTAGTTCATACCAG-----------TCAGTCTTATGGCAGCAGCCCCCATAGACGTATGGGT
marmoset          ACTAGTTCATACCAGCACTAGTTCATACTGTCTTATGGCAGCAGTCCCCATAGATGT------
lemur             ACCGATTCTTACCAG---------------TCTGGTGGCAGCAGCCCCCATAGATGTA-----
bushbaby          ACCAATTCATACCAG---------------TCTGATGGCAGCAGCTCCTAGAGATATAGGGGT
pig               TCTACTCCATACCCG---------------TCTGATGGCAGCAGCCCCCAGACGTGGATGCAT
alpaca            ATTAATTCATACCAG---------------TCGGATGGCAGCAGCCCCCAGAGATGTATAGGT
minke whale       AGTAATTCGTACCAG---------------TCTGATGGCAGCAGCCCCCAGAGAAGTAGGGGT
sheep             AGTAATTCCTACCAG---------------TCTGATGGCAGCAACCCCCGGAAATGTTGGGGT
cow               AGTAATTCATACCAG---------------TCTGATGGCAGCAGCCCCCGGAAATGTTGGGGT
white rhino       ACTAGTTCATACCAG---------------TCTGATGGCAGCAG-CCCCCGTAGATGTATGGG
ferret            ACTAATTTATACTCAC--------------TCTGATGGCAGCAG-CCCCCAGAGATATATGGG
dog               ACTAATTTATACTCG---------------TCTGATGGCAGCATTCCCCAGACATGTATGGGT
panda             ACTGATTTCTACTCA---------------TCTGATGGCAGCAGCCCCCAGGATATATGGGTT
megabat           AATGATTCATGCCAG---------------TCTGATGGCAGCAGCCCCCAGAGATGTAGGGGG
elephant          ----------------------------------ATGGCAGCAGCCTCTAGGGGTGTGAGGGG
                  :  . *   *.* ..               **  .*******.  *      . .


rs835492 alignment
human             GAGATTCTG-ATAGTCACAGCCC----TCAGCAAACTGTTCT-----CTTT-GGAACCAATACA
chimp             GAGATTCTG-ATAGTCACAGCCG----TCAGTGAACTGTTCT-----CTTT-GGAACCAATACA
gorilla           GAGATTCTG-ATAGTCACAGCCC----TCAGCGAACTGTTCT-----CTTT-GGAACCAATACA
orang-utan        GAGATTCCG-ATAGTCACAGCCC----TCAGCGAACTGTTCT-----CTTT-GGAACCAATACT
gibbon            GAGATTCTG-ATAGTTACAGCCC----TCAGCGAACTCTTCT-----CTTT-GGAACCAATACA
rhesus monkey     GAGATTCTG-GTAGTCACAGCCC----TCAGCAAACTGTTCT-----CTTT-GGAACAAATACA
baboon            GAGATTCTG-GTAGTCACAGCCC----TCAGCAAACTATTCT-----CTTT-GGAATAAATACA
squirrel monkey   GAGATTCTG ATAGTCACAGCCC----TCAGCGAACTGTTCT-----CTTT-GGAACCAATA--
lemur             TGGATTCTGGGAATTCGCAGCCAGAATTCTGCAAACTCTTCTTCTCTCTTT-GGAATCAATACA
bushbaby          TAGATTCTGGGTATTCACAGGCC----TCAGCAAACTGTTCTTTTTTTTCC-GGAATCAATAGA
pig               AGGATTCCGGGGATTTATAACTC----TCCAGGAACTGCCCT--CCTTTTT-GGAATCAATACA
alpaca            TGGATTCTGGGGATTTATAGCCC----TCCATGTACTGTCCT--CCTTTTT-GGAATCAACACA
minke whale       TGGATTGTGGGGATTTCTAGCCC----TCCATGAACTGTCCT--CCTTTTT-GGAACCAGTACA
sheep             CGGATTCTGGGAGTTTCTAGCCC----TCCATATACTGTCCT--CCTTT--TGGAATCATTACA
cow               CGGATTCTGGGAGTTTCTAGCCC----TCCATAAACTGTCCT--CCTTTTT-GGAATCAATACA
white rhino       TGGATTCTGGGGATT-ACAGCCT----TCCATGAACTGTTCT-----CTTT-AGAGTCAATACA
ferret            TGGATTCTGGGAATTTGCAGCCC----TCCCTGAACTGTTCT--CTTCTTT-GGAATCGATACA
dog               TGGAGTCTGGGAATTTACAGTCC----TCCGTGAACTGTTCT--CTTCTTT-GGAATTAATACA
panda             TGGATTCTGGGAACTTACAGCCC----TCCAAGAACTGTTCT--CTTCTTTTGGAACCAATACA
                   .** *  * . . *   *.       **   .:***   **      *   .**.  .  *
```

rs835493 alignment

```
human            CACAGAGAAGACCCAGGCAACTCTGGGTTCCAGAAATGAAGGCCTCCT-TGAGAAGCCACAT
chimp            CACAGAGAAGACCCAGGCAACTCTGGGTTCCAGAAATGAAGGCCTCCT-TGAGAAGCCACAT
gorilla          CACAGAGAAGACCCAGGCAACTCTGGGTTCCAGAAATGAAGGCCTCCT-TGAGAAGCCATAT
orang-utan       CACAGAGAAGACTCAGGCAACTCTCGGTTCCAGAAATGAAGGCCTCTT-TGAGAAGCCACAT
gibbon           CACAGAGAAGACCCAGGCAGCTCTGGGTTCCAGAAATGAAGGCCTCCT-TGAGAAGCCACAT
rhesus monkey    CACAGAGAAGACCCAGGCAATGCTGGGTTCCAGAAATGAAGGCCTCCT-TGAGAAGCCACTT
baboon           CACAGAGATGACCCAGGCAATGCTGGGTTCCAGAAATGAAGGCCTCCT-TGAGAAGCCACTT
squirrel monkey  CACAGAGAAGACTCAGGCAACGCTGGGTTCCAGAAACGAAGGGTTCCT-TGAGAAGCCACTT
marmoset         ------------------------------CAGAAATGAAGGCCTCCTCTGAGAATCCCACA
bushbaby         ----------------------CTGGGTTCCAGAAATGCAGGCAGCCCCCG-GCAGCAAGTT
lemur            CACAGAGAAGCCCCAGGCAATGCTGGGTTCCAGAAATG------------------------
pig              -ACAGAGAAGCCCCAGGCAACTC-GGGTTCCAGAAATGAAGGC-------------------
alpaca           ----------CCCCAGGCAACTCTGGGTTCCAGAA---------------------------
minke whale      -----------------------------CCAGAA---AAGGCCTCCT-TGAGAAGC------
dolphin          -----------------------------CCAGAA---AAGGCCTCCT-TGAGAAGC------
sheep            --------AGACCCAGGCAACTCTGG------------------------------------
cow              ----------------------------------AATGAAGGCCTCCT-TGAGAAG
white rhino      ----------------------CTGGGTTCCAGAAATGAAGGCATCCT-TGGGCAGCCATTG
cat              ----------CCCCAGGCAACTCTGGTTTCCAGAA-TGAAGGTGTCCT-CAAGCAGCCATGG
ferret           TCCAGAGAAGCCCCAGGCAATGCTGAGTTCCAGAAAGGAAGGCGTCCT-CAGGCAGCCATTG
dog              CACAGAGAAGCCCCAGGCAACGCTAAGCTCCAGAAAGGAAGGC-------------------
panda            CCCAGAGAAGCCCCAGGCGACAGTGAGTTCCAGAA---------------------------
```
