# Supplementary figures and images for: Functional Characterization of the Osteoarthritis Susceptibility Mapping to CHST11—A Bioinformatics and Molecular Study
Source: PLoS One. 2016 Jul 8;11(7):e0159024. doi: 10.1371/journal.pone.0159024 (PMC4938163; doi:10.1371/journal.pone.0159024)

**A**

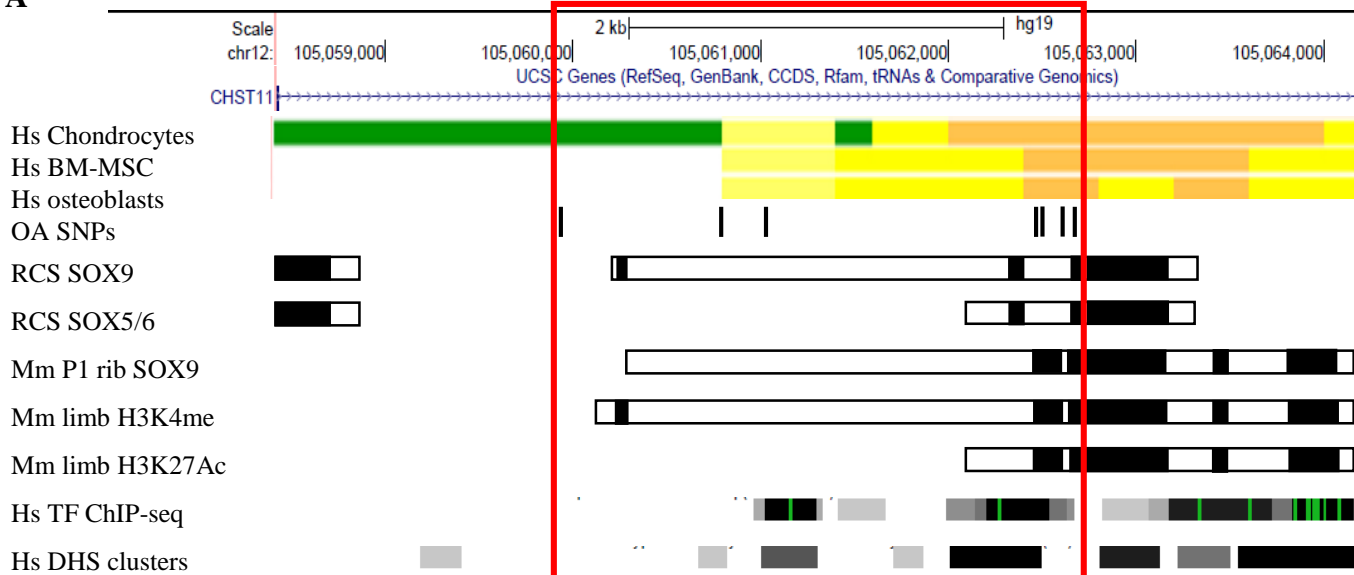

**B**

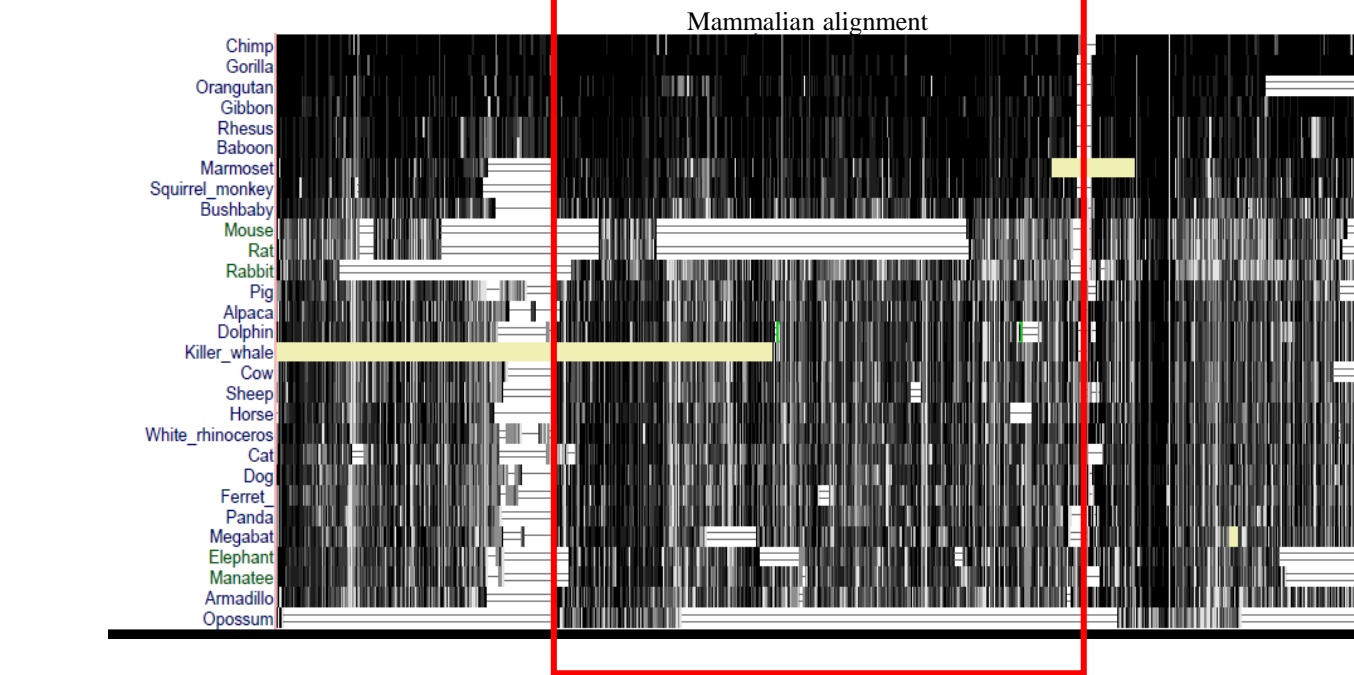

Supplement: S2 Fig — (A) UCSC genome browser view of the LD block and surrounding region. The LD block was defined as the region containing SNPs with r2 of 0.8 or greater with the OA SNP rs835487 within the EUR population and is indicated by the red rectangle. The black rectangles indicate the regions with homology to the SOX9 and SOX5/6 ChIPseq peaks present in rat proliferating/early prehypertrophic growth plate chondrocytes (RCS tracks; Liu and Lefebvre 2015 [16]), the SOX9 ChIPseq peaks in proliferative and prehypertrophic rib chondrocytes from postnatal day 1 mice (Mm P1 rib SOX9; [15]), and the H3K4me1 and H3K27Ac ChIPseq peaks from E14.5 mouse limb (Mm limb H3K4me and Mm limb H3K27Ac respectively). Homologous regions were identified using the UCSC LiftOver tool between the rat RGSC 5.0/rn5, the mouse NCBI37/mm9 and the human GRCh37/hg19 assemblies. The filled black rectangles within the homologous blocks represent the regions with over 70% identity between the human and rat/mouse nucleotide sequences; these regions were identified using the blastn sequence alignment tool that is optimised for ‘somewhat similar sequences’. The chromatin state segmentation tracks for human bone-marrow derived mesenchymal stem cells (Hs BM-MSC), in vitro differentiated chondrocytes (Hs Chondrocytes) and primary osteoblasts (Hs osteoblasts) from the RoadMap Epigenome project, human transcription factor binding sites (Hs TF ChIP-seq) and DnaseI hypersensitivity sites (Hs DHS clusters) from the ENCODE project, and human enhancer transcripts from the FANTOM 5 consortium (Hs transcribed enhancer; [26]) are also indicated. The seven vertical lines mark the positions of, from left to right, rs835486, rs835487, rs835488, rs835490, rs835491, rs835492 and rs835493 (OA SNPs). (B) Alignment of the LD region in 29 mammalian species generated using the Multiz tool in UCSC. (PDF) [file pone.0159024.s002.pdf]

**A**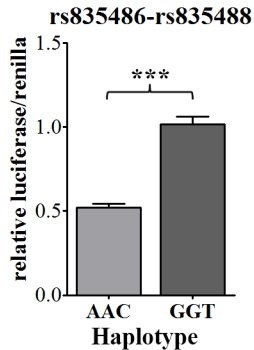**B**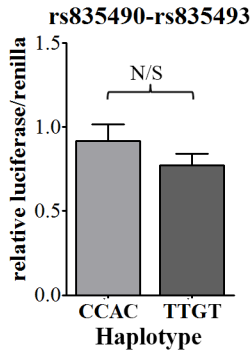**C**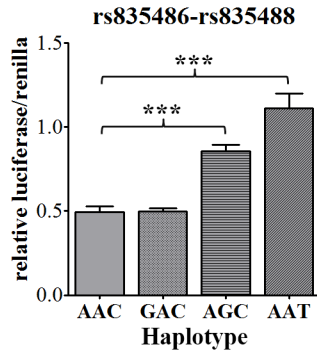**D**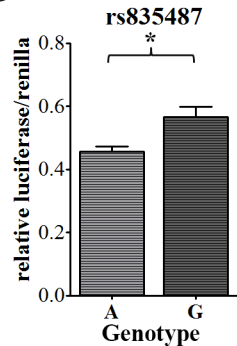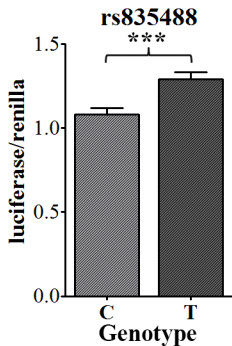

Supplement: S3 Fig — (A) to (D) Luciferase enhancer assays in the MDA-MB-231 human adenocarcinoma cell line using the pGL3 promoter plasmids. The vectors were co-transfected with the pRL-TK Renilla plasmid, and luciferase/renilla values normalised to the empty pGL3-enhancer vector. (A) Luciferase activity of the AAC and OA-associated GGT haplotype of the region containing the rs835486, rs835487 and rs835488 SNPs. (B) Luciferase activity of the CCAC and OA risk TTGT haplotype of the region containing rs835490, rs835491, rs835492 and rs835493. (C) Luciferase activity of the rs835486-rs835487-rs835488 vectors containing the AAC, GAC, AGC and AAT haplotypes. Mutating the non-OA allele into the OA-risk allele of rs835486, rs835487 or rs835488, respectively, created the GAC, AGC and AAT vectors. (D) Luciferase activity of the two alleles of rs835487 only (left) or rs835488 only (right). There is increased luciferase activity of the OA associated alleles of both SNPs. Data shown is the mean ± standard error of at least four independent experiments, each with five technical repeats. *p < 0.05, ***p < 0.001, Mann-Whitney U test. N/S, not significant. The data points that enabled construction of this figure can be found in S5 Table. (PDF) [file pone.0159024.s003.pdf]

**A****rs835487 A allele supershifts**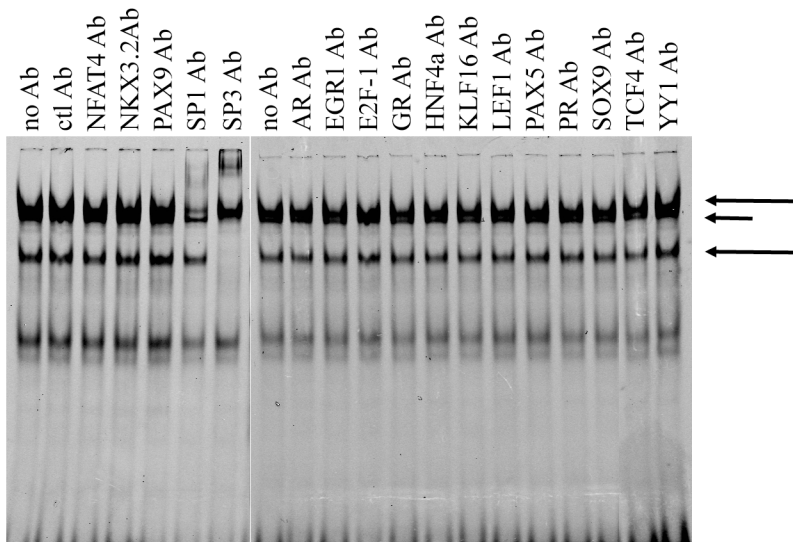**B****rs835488 C allele supershifts**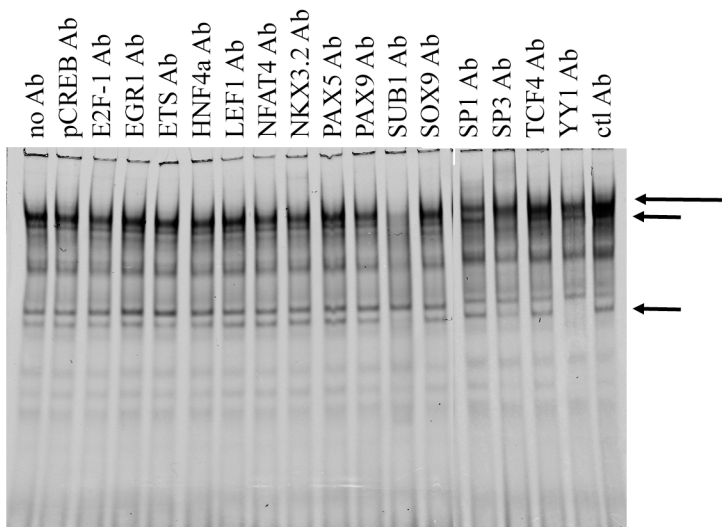

Supplement: S5 Fig — (A) rs835487 A allele gel supershift assays with antibodies against 17 transcription factors. The rs835487-protein complexes are only supershifted upon addition of antibodies against SP1 or SP3, with no supershifts observed for the other 15 antibodies tested. The SP1-containing complexes are indicated by the long arrows, with the short arrow indicating SP3-containing complexes. (B) rs835488 C allele supershift assays with antibodies against 16 transcription factors. Addition of an anti-SP1 antibody reduces formation one of the rs835488-protein complexes (long arrow), and formation of two complexes are decreased upon the addition of anti-SP3 or anti-YY1 antibodies (short arrows). An anti-SUB1 antibody reduces formation of all three SP1/SP3/YY1 containing complexes. Nuclear extracts from SW1353 human chondrosarcoma cell line were used in all EMSAs. Ab, antibody. Ctl Ab, anti-PAX6 polyclonal antibody used as an IgG control. (PDF) [file pone.0159024.s005.pdf]

**A**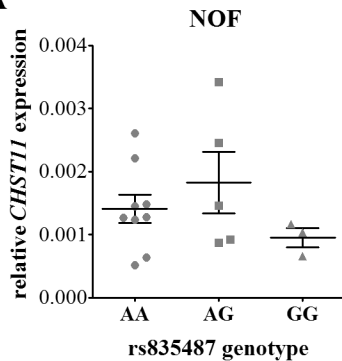**B**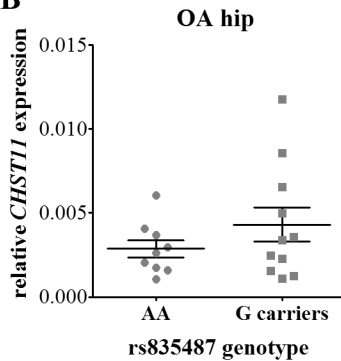**C**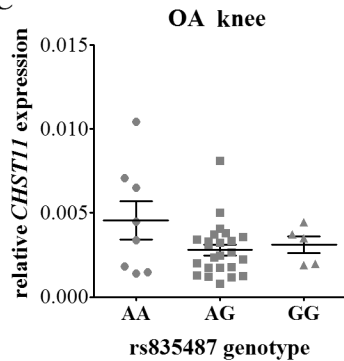**D**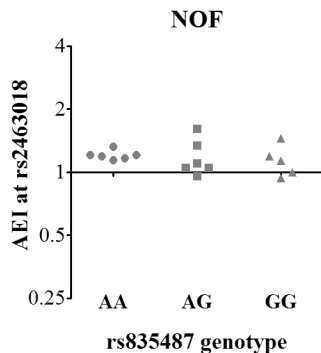**E**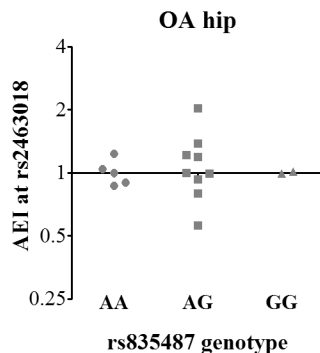**F**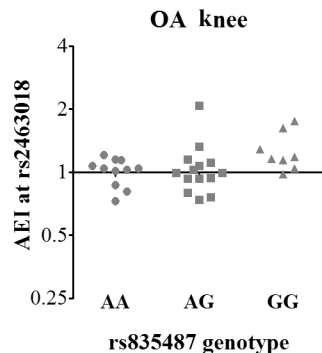

Supplement: S6 Fig — (A) to (C) CHST11 expression in (A) neck of femur (NOF) fracture, (B) OA hip and (C) OA knee cartilage stratified by genotype at the OA-associated SNP rs835487. Expression was measured by qRT-PCR and normalised to the housekeeping genes 18s, HPRT1 and GAPDH. The error bars indicate the mean ± standard error. Due to the low number of GG homozygotes in the OA hip analysis, the heterozygotes and GG homozygotes were combined as G carriers and compared to the AA homozygotes. (D) to (F) Allelic expression imbalance (AEI) of the CHST11 transcript SNP rs2463018 stratified by genotype at the OA SNP rs835487 in (D) NOF, (E) OA hip and (F) OA knee cartilage. Although only rs835487 genotypes are shown, rs835487 and rs835488 had an r2 of 1 in these 66 individuals, with the AA, AG and GG genotypes at rs835487 being in complete LD with the CC, CT and TT genotypes at rs835488 respectively; rs835487 and rs835488 genotypes are therefore directly interchangeable in this data set. Allelic ratios were normalised to cartilage DNA from the same individual. For (A) to (F), each circle, square and triangle represents data from a single individual. (PDF) [file pone.0159024.s006.pdf]
