# Supplementary material for: Functional Characterization of the Osteoarthritis Susceptibility Mapping to CHST11—A Bioinformatics and Molecular Study
Source: PLoS One. 2016 Jul 8;11(7):e0159024. doi: 10.1371/journal.pone.0159024 (PMC4938163; doi:10.1371/journal.pone.0159024)

A

rs835487 A allele probe TGGCAAATAGGAGGT**A**CCTTTAAAACGCTTG

rs835487 G allele probe TGGCAAATAGGAGGT**T**CCTTTAAAACGCTTG

B

rs835487 competition EMSA

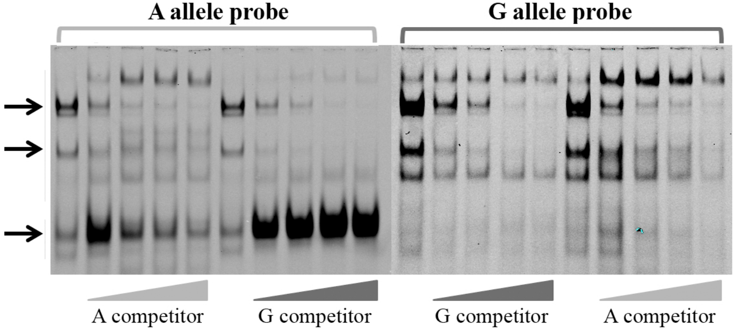

C

rs835488 C allele probe TAGATTCTTCCAGGC**C**GTCTCATTAGAAGTT

rs835488 T allele probe TAGATTCTTCCAGGC**T**GTCTCATTAGAAGTT

D

rs835488 competition EMSA

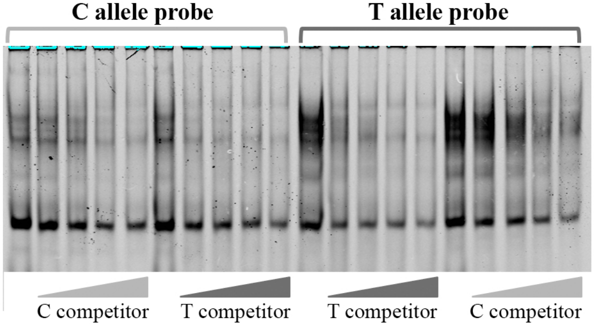

Supplement: S4 Fig — (A) Positive strand sequence of the rs835487 double-stranded EMSA DNA probes with the alleles of the SNP highlighted in bold. (B) MDA-MB-231 nuclear lysate EMSA with rs835487 A and G allele probes with and without unlabelled A and G allele competitors. Unlabelled competitors were present in 0-, 10-, 25-, 50- and 100-fold molar excess as indicated and arrows denote rs835487-protein complexes. (C) Positive strand sequence of the rs835488 probes with the alleles of the SNP highlighted in bold. (D) rs835488 EMSA with the C and T allele probes with 0-, 10-, 25-, 50- and 100-fold molar excess of unlabelled C and T allele competitors and MDA-MB-231 nuclear proteins. (PDF) [file pone.0159024.s004.pdf]
