# Supplementary material for: Functional Characterization of the Osteoarthritis Susceptibility Mapping to CHST11—A Bioinformatics and Molecular Study
Source: PLoS One. 2016 Jul 8;11(7):e0159024. doi: 10.1371/journal.pone.0159024 (PMC4938163; doi:10.1371/journal.pone.0159024)
Supplement: S1 Table — The bold and underlined nucleotides in the Site directed mutagenesis primers are the mutagenized SNP alleles; the bold and underlined nucleotides in the EMSA probes are the SNP sites; the underlined sequences in the SP1/SP3 primers is the consensus sequence for the transcription factors. (PDF) [file pone.0159024.s007.pdf]

The primers used in this study

Method	Primer sequence (5'-3')
**Cloning into pGL3 promoter**	
rs835486-rs835487-rs835488	MluI FP GGGGACGCGTCCCGGCCTCTTTCTCTTTTAAT
	XhoI RP GGGGCTCGAGCTGTCCCTGATTGAGATG
rs835490-rs835491-rs835492	KpnI FP GGGGGGTACCTCCGTATGTGGAGCATCTCTGT
	NheI RP GGGGGCTAGCCATTTGCACCATTTCTGAGAGC
rs835486 only	MluI FP GGGGACGCGTCGGCCTCTTTCTCTTTTAATCTT
	XhoI RP GGGGCTCGAGAGCTCTACTGTTCCACACAGGAC
rs835487 only	MluI FP GGGGACGCGTTTTCCTTCCGTTGTTCTTGG
	XhoI RP GGGGCTCGAGAGCTCAGGCCAAATGACTGT
rs835488 only	MluI FP GGGGACGCGTTGAGCAGAACATTCCAGTTGA
	XhoI RP GGGGCTCGAGAAACTGAGGCCCAGAGGAAT
**Site directed mutatgenesis**	
rs835486 A allele	FP TTAGACTATCATGT**A**TAGAATTAAGATTG
	RP CAATCTTAATTCTA**T**ACATGATAGTCTAA
rs835486 G allele	FP TTAGACTATCATGT**G**TAGAATTAAGATTG
	RP CAATCTTAATTCTA**C**ACATGATAGTCTAA
rs835487 A allele	FP GGCAAATAGGAGGT**A**CCTTTAAAACGCTT
	RP AAGCGTTTTAAAGG**T**ACCTCCTATTTGCC
rs835487 G allele	FP GGCAAATAGGAGGT**G**CCTTTAAAACGCTT
	RP AAGCGTTTTAAAGG**C**ACCTCCTATTTGCC
rs835488 C allele	FP TAGATTCTTCCAGGC**C**GTCTCATTAGAAGTT
	RP AACTTCTAATGAGAC**G**GCCTGGAAGAATCTA
rs835488 T allele	FP TAGATTCTTCCAGGC**T**GTCTCATTAGAAGTT
	RP AACTTCTAATGAGAC**A**GCCTGGAAGAATCTA
**DY-682 EMSA probes**	
rs835487 A allele	FP TGGCAAATAGGAGGT**A**CCTTTAAAACGCTTG
	RP CAAGCGTTTTAAAGG**T**ACCTCCTATTTGCCA
rs835487 G allele	FP TGGCAAATAGGAGGT**G**CCTTTAAAACGCTTG
	RP CAAGCGTTTTAAAGG**C**ACCTCCTATTTGCCA
rs835488 C allele	FP AGATTCTTCCAGGC**C**GTCTCATTAGAAGT
	RP ACTTCTAATGAGAC**G**GCCTGGAAGAATCT
rs835488 T allele	FP AGATTCTTCCAGGC**T**GTCTCATTAGAAGT
	RP ACTTCTAATGAGAC**A**GCCTGGAAGAATCT
**SP1/SP3 consensus competitor**	FP: 5′-AATTGGGGGGGCGGGGGTACGTAGCA-3′
	RP: 5′-TGCTACGTACCCCCGCCCCCCCAATT-3′
**Genotyping**	
rs835487 - A allele cut by KpnI	FP GGAGTGAGCCAGAACAGATAAG
	RP CGTGAATTTGGTCCAGCAGAG
rs2463018 - cut by BccI	FP GGTGAGACTCGTACACTTGG
	RP CTAGCTGTAAGAGGGATGTGG
**Allelic expression imbalance (AEI)**	
Pyrosequencing	FP btn CATGCACACCCTCTCACG
	RP GGGGCATGGATTATGGAA
	seq GCAGTTACCAAGATGCTGA
**qRT-PCR**	
CHST11	FP TGCTTGGGATCCTTTATCCTG
	RP GCTGGATTGGGTTGTAGAGTTC
	probe AATCCCTTTGGTGTGGACATCTGCT
18s	FP CGAATGGCTCATTAAATCAGTTATGG
	RP TATTAGCTCTAGAATTACCACAGTTATCC
	probe TCCTTTGGTCGCTCGCTCCTCTCCC
GAPDH	FP ACATCGCTCAGACACCATG
	RP TGTAGTTGAGGTCAATGAAGGG
	probe AAGGTCGGAGTCAACGGATTTGGTC
HPRT1	FP TGCTGAGGATTTGGAAAGGG
	RP ACAGAGGGCTACAATGTGATG
	probe AGGACTGAACGTCTTGCTCGAGATG
