# Supplementary material for: Functional Characterization of the Osteoarthritis Susceptibility Mapping to CHST11—A Bioinformatics and Molecular Study
Source: PLoS One. 2016 Jul 8;11(7):e0159024. doi: 10.1371/journal.pone.0159024 (PMC4938163; doi:10.1371/journal.pone.0159024)
Supplement: S3 Table — The consensus and competitor sequences used in the subsequent competition EMSAs are also listed. (PDF) [file pone.0159024.s009.pdf]

rs835487 transcription factors

Transcription factor	Sequence of probe bound	Competitor sequence forward	Competitor sequence reverse
ABI4	GGTA/GCC	TAGCACCTGACGGCACCCTGAGTACGTAG	CTACGTACTCAGGGTGCCGTCAGGTGCTA
EVE/evx1/evx2	A/GCCTTTAAA	TAGCACCTGACTGAAAAATTAAACGTAG	CTACGTTTAATTTTTCAGTCAGGTGCTA
HNF3beta	A/GCCTTTA	TAGCACCTGACTGAATATTTATACGTAG	CTACGTATAAATATTCAGTCAGGTGCTA
KLF	AAAGG	TAGCACCTGACTGACCCTTTATACGTAG	CTACGTATAAAGGGTCAGTCAGGTGCGT
LF-A1	TGCCTTT	TAGCACCTGACTGTGCCCTGGTACGTAG	CTACGTACCAGGGCACAGTCAGGTGCTA
MSX1	GTG	TAGCACCTGACTGTGTCTGAGTACGTAG	CTACGTACTCAGACACAGTCAGGTGCTA
NFAT4 (NFATc3)	GCCTTTAA	TAGCACCTGACTGAGAATTTGAACGTAG	CTACGTTCAAATTCTCAGTCAGGTGCTA
NF-BA1	ACCTTT	TAGCACCTGACTGAACCTTTGTACGTAG	CTACGTACAAAGGTTCAGTCAGGTGCTA
NKX3.2	TAGGAGG	TAGCACCTGGAGGCGTCTGAGTACGTAG	CTACGTACTCAGACGCCTCCAGGTGCTA
PAX9	GGAGGTG	TAGCACCTATCGTGGTCTGAGTACGTAG	CTACGTACTCAGACACCGATAGGTGCTA
PLAG	ATAGGAGGTACCT	TAGCAATGGGGGGTACCATAGTACGTAG	CTACGTACTATGGTACCCCCCATTGCTA
SP1/SP3	GGAGGTGCC	AATTGGGGGGGCGGGGGTACGTAGCA	TGCTACGTACCCCCGCCCCCCCAATT
TATA	CCTTTAAAAC	TAGCACCTGACTGACCTATAAAAGGTAG	CTACCTTTTATAGGTCAGTCAGGTGCTA
ZIC1	A/GCCT	TAGCACCTGAGACCACCCAGAGTACGTAG	CTACGTACTCTGGGTGGTCTCAGGTGCTA

rs835488 transcription factors

Transcription factor	Sequence of probe bound	Competitor sequence forward	Competitor sequence reverse
AP-2alphaA	CCAGGC	TAGCACCTCCAGGCCCGTCTGAGTACGTAG	CTACGTACTCAGACGGGCCTGGAGGTGCTA
AP1	C/TGTCTCA	TAGCACCTGCTGATGTCTCAGTACGTAC	CTACGTACTGAGACATCAGTCAGGTGCTA
CREB	GCTGTCT	TAGCACCTGACTGCTGTCTGAGTACGTAG	CTACGTACTCAGACAGCAGTCAGGTGCTA
HOXA3	CTGTC	TAGCACCTGACTGCTGTCTGAGTACGTAG	CTACGTACTCAGACAGCAGTCAGGTGCTA
PAX5	GTCTC	TAGCACCTGACTGACGTCTCAGTACGTAG	CTACGTACTGAGACGTCAGTCAGGTGCTA
POU2F1 (Oct-1)	GTCTCATTAGA	TAGCACCTGACTGACGTCTCATTAGATAG	CTATCTAATGAGACGTCAGTCAGGTGCTA
SP1/SP3	CCAGGCCGTC	AATTGGGGGGGCGGGGGTACGTAGCA	TGCTACGTACCCCCGCCCCCCCAATT
TCF3/E47	TCCAGGC	TAGCACCTCCAGGCTGTCTGAGTACGTAG	CTACGTACTCAGACAGCCTGGAGGTGCTA
TFAP2A	CCAGGC	TAGCACCTCCAGGCCCGTCTAGTACGTAG	CTACGTACTAGACGGGCCTGGAGGTGCTA
