# Supplementary material for: Functional Characterization of the Osteoarthritis Susceptibility Mapping to CHST11—A Bioinformatics and Molecular Study
Source: PLoS One. 2016 Jul 8;11(7):e0159024. doi: 10.1371/journal.pone.0159024 (PMC4938163; doi:10.1371/journal.pone.0159024)
Supplement: S4 Table — Details of the gene transcription start site (TSS) and function of the gene/encoded protein are listed, together with information on whether they demonstrate altered expression in OA cartilage versus non-OA cartilage (using data from [32]), or whether they show altered expression in OA cartilage versus non-OA cartilage (using data from [22,33]). Also listed is whether they have a reported role in the musculoskeletal system based on searches of PubMed (http://www.ncbi.nlm.nih.gov/pubmed) and OMIM (http://www.ncbi.nlm.nih.gov/omim) and information from mice deletion models. (PDF) [file pone.0159024.s010.pdf]

Genes with transcription start sites located within the 2Mb region surrounding rs835487. TSS; transcription start site.

Gene	TSS	Gene/protein function	Altered expression in OA cartilage?	Altered methylation in OA cartilage?	PubMed	OMIM	Mouse model
NT5DC3	104,234,975	pancreatic cancer	no	no	no	N/A	NA
GNN	104,324,039	pseudogene	no	no	no	N/A	N/A
HSP90B1	104,324,112	molecular chaperone involved in processing and transport of secreted proteins	no	no	no	N/A	-/- mice die at 7dpp, conditional KO has gut phenotype
C12orf73	104,359,473	conserved protein of unknown function	no	no	no	N/A	N/A
TDG	104,359,593	DNA glycosylase involved in DNA demethylation and DNA repair	no	no	no	N/A	-/- mice die at e12.5
MIR3652	104,324,333	microRNA, involved in adiopogensis?	no	no	no	N/A	N/A
GLT8D2	104,457,961	glycosyltransferase, regulates ApoB100 in hepatocytes, associated with liver fibrosis	yes, up in hip OA	no	no	N/A	N/A
HCFC2	104,458,236	transcriptional co-activator, involved in HSV viral infection	no	no	no	N/A	N/A
NFYB	104,532,040	transcription factor that binds CCAAT motifs.	no	no	no	N/A	N/A
TXNRD1	104,680,460	thioredxin reductase, plays role in selenium metabolism & protecting against oxidative stress	yes; up in knee OA	no	knock-down has no effect on chondrocytes	N/A	mice die at e9.5, fail to gastrulate
EID3	104,697,510	tissue specific component of SMC5/6 DNA repair complex, transcriptional repressor	no	no	no	N/A	N/A
CHST11	**104,850,692**	**sulfotransferase that sulfates chondroitin, the predominant proteoglycan in cartilage**	**yes; up in knee OA**	**yes**	**chondrocyte development, growth plate morphogenesis**	N/A	**-/- mice have severe chondrodysplasia**
MIR3922	104,985,494	microRNA	no	no	no	N/A	N/A
SLC41A2	105,322,472	plasma membrane magnesium transporter	no	no	no	N/A	N/A
C12orf45	105,380,098	conserved protein of unknown function	no	no	no	N/A	N/A
ALDH1L2	105,478,341	mitochondrial aldehyde dehydrogenase with formyltetrahydrofolate dehydrogenase activity	no	no	no	N/A	N/A
KIAA1033	105,501,492	component of WASH complex involved in actin polymerisation and intracellular transport	no	no	no	MRT43; 615817	N/A
APPL2	105,630,008	required for regulation of cell proliferation in response to extracellular signals	no	yes	no	N/A	N/A
C12orf75	105,724,414	conserved protein of unknown function, upregulated in a subset of colon cancers	no	no	no	N/A	N/A
